# Supplementary material for: A frog with three sex chromosomes that co-mingle together in nature: Xenopus tropicalis has a degenerate W and a Y that evolved from a Z chromosome
Source: PLoS Genet. 2020 Nov 9;16(11):e1009121. doi: 10.1371/journal.pgen.1009121 (PMC7652241; doi:10.1371/journal.pgen.1009121)
Supplement: S1 Text — (PDF) [file pgen.1009121.s002.pdf]

# S1 Text

## Supplemental Methods

### Identifying Putative Genotype Errors in Prior to Sex-Linkage Analysis

Genotyping errors inflate linkage map lengths, and reduce power to infer genotype–phenotype associations. These errors can be caused by incomplete digestion of DNA during RRGS library preparation, polymorphism at restriction enzyme cut sites, and low or allelic bias in sequencing coverage [1–3]. We attempted to reduce the effects of genotyping errors by inspecting three consecutive offspring genotypes in up to a 5 Mb window, resolving the sex-specific parental haplotypes in each window, and setting offspring genotypes to missing data that could only be explained by a double recombination event, under the assumption that double recombination events are unlikely within 5 Mb windows.

To accomplish this, we first assigned variants in offspring genotypes to each parent. For example, if the genotype of an offspring was T/A, the mother was A/T, and the father was A/A, we can infer that the T was maternally inherited and that the A was paternally inherited. Then, in groups of three markers spanning no more than 5 Mb (and spaced at least 5 Kb apart from each other, randomly discarding extra SNPs within 5 Kb windows), we scored the frequencies of multilocus haplotypes that were inherited from each parent. We assumed that the non-recombined haplotypes would be more frequent in the offspring than recombined haplotypes, and thus reflect the parental gametic phases. For example, the phases of a set of three markers might be inferred to be ACT/TGC in the mother, and TGC/TGC in the father. In the offspring, we then identified non-recombined haplotypes (ACT, TGC), single recombination haplotypes (AGC, TCT, ACC, TGT), and double recombination haplotypes (AGT, TCC).

It is possible that setting suspected genotype errors to missing data could lead to underestimated map lengths by removing any real double recombination events. However, these events are probably infrequent and the effect therefore is expected to be modest. As well, our interest is primarily in comparing map lengths between the sexes, and we treated both maternal and paternal sets of markers identically, so any underestimation of recombination rates is likely to similarly affect both.

## Supplemental Results

In the Ghana west family Family 1 after FDR correction, five paternal SNPs had a significant association with offspring sex; all of these were located on one end of chromosome 7. Genome-wide heterozygosity was similar in the parents of Family 1, which suggests that the power to detect female-linked and male-linked variants is similar. 2,857 sites were heterozygous in the father, 2,834 were heterozygous in the mother, and 1,660 were heterozygous in both parents. In Ghana east Family 2, three paternal SNPs were identified that were significantly associated with offspring sex, but none of these were significant after FDR correction (this cross included far fewer offspring; Fig. 4). These three SNPs were in a similar locations (within 1.6 Mb) to the sex-linked sites in Family 1 (Fig. 4). Other chromosomes (Fig. S3) did not have any variants with a significant association with sex for Family 1, but in Family 2 there were putative false positives (prior to FDR correction) on chromosomes 3 and 5 (Fig. S3). Genome-wide, there were 2447 paternal heterozygous SNPs, 2700 maternal heterozygous SNPs, and 2197 sites that were heterozygous in both parents of Family 2.

In the Ghana west Family 1, one of the five significantly sex-linked SNPs (at position 9,149,465 in *X. tropicalis* genome assembly version 10 (v10) was also 100% sex-linked (heterozygous in all sons, homozygous in all daughters), and the variants near this site (positions 8,104,283 – 13,579,674) were very strongly, but not completely sex linked. In Ghana east Family 2, all three of the significantly sex-linked SNPs were 100% linked with sex (positions 2,679,758, 5,227,940, 6,542,981). Differences in $p$-values between these three SNPs in Fig. 4 are a consequence of differing numbers of missing offspring genotypes. The distinctive statistical signatures of sex-linkage between the Ghana west Family 1 and Ghana east Family 2 (significant after FDR correction for the former, but not for the latter) is a consequence of the different number of offspring analyzed (Fig. 2).

## Sex-linked regions in *Xenopus mellotropicalis*

A closely related allotetraploid species, *X. mellotropicalis*, has almost completely female-linked variation in the *or8h1* gene [4]. This locus maps to position 11,962,496–11,963,464 on chromosome 7 in v10 (3,679,275 – 3,680,243 in v9), which is very close to the third most strongly female-linked genomic region in *X. tropicalis* (linkage group super_352:0; positions 3,272,993 – 3,436,880 in v9; LOD score: 12.60676032) [5]. Similarly, a scaffold from the genomee of *X. mellotropicalis* with a completely sex-linked SNP corresponds to positions 250,772 —251,327 on chromosome 7 in v10 (scaffold_622, positions 39,157 – 38,626 in v9). Both of these female-linked variants in *X. mellotropicalis* are homologous to portions of the the 95% Bayes credible interval (positions 0 – 3,906,563 in v9) for the W-linked female determining marker in *X. tropicalis* [5]. As discussed in the Introduction, this supports the proposal that female heterogamy is a shared ancestral condition in *X. tropicalis* and *X. mellotropicalis*, and that the Y chromosome of *X. tropicalis* is younger than and derived from the W or Z chromosomes in this species.

## Pairwise nucleotide polymorphism in expressed transcripts

We initially expected WW offspring to have no polymorphism in sex-linked transcripts because both of these W chromosomes descend from specimen BJE4362, who was the father and the grandfather of offspring of Family 3 (Fig. 2), but this expectation was not borne out (Fig. 5). We suspect that this is a consequence of mapping error, for example due to repetitive regions in untranslated regions of these transcripts. Another non-exclusive possibility is that recombination occurred in the distal portion of the sex-linked region of the W and Z chromosomes during oogenesis in the mother or in distal portion of the sex-linked region of the W and Y chromosomes during spermatogenesis in the father/grandfather. To test these possibilities, we separately quantified polymorphism in transcripts encoded by genes <6 Mb on chromosome 7 in v10 and between 6 Mb and 11 Mb of the sex-linked region (Fig. S4). If recombination occurs between the W and Y sex chromosomes (in the father of Family 3) or between the W and Z chromosomes (in the mother of Family 3), we expected diversity of the first half would be similar to levels seen in other recombining regions of the genome (i.e. the pseudoautosomal and autosomal regions). However, within three of the four sex chromosome genotypes, the first portion and second portions of the sex-linked region had similar diversities and these diversity levels were generally not similar to the pseudoautosomal and autosomal regions (see below). In the putative ZY individuals, the diversity in the 5' portion of the sex-linked region was similar to that in the autosomal region. We suspect that recombination between the Z and Y chromosomes causes polymorphism to be similar in the first half of the sex-linked region. We therefore favor a technical explanation for the inference of polymorphism in these

putative WW individuals over the biological explanation associated with recombination between W chromosome and the Y or Z chromosomes (Fig. 5).

We had no expectation about the relative level of pairwise nucleotide polymorphism in expressed transcripts in recombining compared to non-recombining regions because we do not know the proportion of sex-linked transcripts that are only expressed on one sex chromosome (and thus completely homozygous, which decreases polymorphism compared to expressed non-sex linked transcripts) or the level of divergence between expressed transcripts on different sex chromosomes (which generates heterozygous genotypes and increases polymorphism compared to expressed non-sex linked transcripts). Moreover, the level of divergence between non-recombining portions of sex chromosomes need not be constant throughout the non-recombining region [6]. The average polymorphism of expressed non-sex-linked transcripts was 0.013 (range: 0.012-0.015). That this level of polymorphism is lower than that of expressed sex-linked transcripts in putative WZ females and WY males argues for the presence of diverged sites between the sex-linked regions of the W and Z chromosomes and the W and Y chromosomes.

## Reference Bias

One possible concern for these analyses relates to the possibility that reference bias – when the reference sequence influences genotypes or mapping of transcripts – could affect our results. The effect of reference bias is typically evidenced by poor mapping or inferences of lower expression in the heterogametic sex when data are mapped to a reference genome from the homogametic sex (e.g., [7]). In the current study, however, expression levels were assessed using a transcriptome assembly we generated *de novo*, and the genomic locations of assembled transcripts was assessed by mapping these transcripts to the genome assembly from a female individual that may have been heterogametic (WZ) or homogametic (WW). In several analyses using the entire data sets or subsets with specific sex chromosome genotypes, we consistently observed an excess of male-biased expression of sex-linked transcripts in heterogametic males compared to females (Figs. S6, S7, S8). For these reasons we believe our inference of extensive male-biased expression of transcripts in the sex-linked region has a biological rather than a technical basis.

Because the *X. tropicalis* individual that was used for genome sequencing was female, Y chromosome specific sequences or genes, might not be present in this genome sequence. We therefore searched for transcripts with male-specific expression in the tadpole gonad/mesonephros transcriptome that did not have a strong mapping to v10. Only one unmapped transcript had substantial male-specific expression (TRINITY_DN3464_c0_g2_i2). However, we were able to amplify and sequence a portion of this transcript in both sexes, which indicates that the gene encoding this transcript is not specific to the Y chromosome, although we did identify a diverged position between one male and one female sequence (several other individuals failed to amplify or sequence). As discussed above, other factors such as GC content and repetitive sequences also may have contributed to mapping error of transcripts to the genome assembly or of RNAseq reads to the transcriptome assembly. However, we have no reason to suspect GC content would disproportionately affect mapping in certain genomic regions (e.g., sex-linked and non-sex-linked) or in a specific sex.

## Expression Bias in Offspring subsets from Family 3

If the inferred sex chromosome genotypes of the parents of Family 3 discussed in the main text are correct, we predicted that patterns of male-biased expression might be distinctive if we reanalyzed the data using a subset of individuals (subset 1) that

included only putative WW females (XT3, XT9, XT11, XT20) and only putative WY males (XT7, XT8) as compared to a subset (subset 2) that included only putative WZ females (XT2, XT6, XT10, XT16, XT17) and only putative ZY males (XT1, XT13, XT19) because females do not have a Z chromosome in the first subset but they do in the second subset. As expected, more significantly male-biased transcripts were identified in the analysis of subset 1 (Fig. S6) than subset 2 (Fig. S7), even though subset 1 included fewer individuals of both sexes. Another interesting finding that emerges from these analyses is that the portion of chromosome 7 with the high density of genes with sex-biased expression extends slightly beyond the sex-linked region that we identified when all individuals were included for subset 1, but not for subset 2. This suggests that the degeneration of the W chromosome may extend beyond 11Mb, but the male-biased expression associated with this degeneracy on the W chromosome in this region is not as apparent in WZ females due to the presence of the Z chromosome.

When we performed an analysis of differential expression between putative WW females and putative ZY males (subset 3), the results were similar to the subset 1 analysis in terms of number and identities of differentially expressed transcripts in and near the sex-linked region (Fig. S8). We also performed an analysis of only putative WZ females and putative WY males (subset 4). If male-biased expression were solely due to degeneration on the W chromosome, we expected this comparison to have even more modest sex-differences than the other subsets. However, the pattern of male biased expression was relatively similar to the analysis of subset 2 (which is putatively WZ females versus ZY males; Fig. S9). This suggests that, in addition to degeneration of the W chromosome, there may be regulatory differences of alleles on the sex-linked portions of the Z and Y chromosomes that contribute to male-biased expression in the main analysis (Fig. 6). Alternatively or in addition, regulation of several genes in the sex-linked portion of the Z chromosome may vary depending on whether it this chromosome is in a female or male individual.

**Table A.** Genes on chromosome 7 between 0–11 Mb in v10 genome assembly with significantly male or female biased expression in gonad/mesenephros tissue of stage 50 tadpoles. Information includes transcript ID (TransID), the Start and End coordinates in v10, annotation, log male/female fold change (logFC), the *p*-value after false detection rate correction (FDR), and the nature of the biased expression (Bias), whether male or female, and whether or not the transcript was sex-specific (Sex-specific).

| TransID | Start | End | Annotation | logFC | FDR | Bias | Sex-specific |
|---|---|---|---|---|---|---|---|
| TRINITY_DN4289_c0_g1_i1 | 1075164 | 1075454 | add3 (adducin 3) - intronic | 5.388 | 0.012 | male | yes |
| TRINITY_DN3468_c0_g1_i5 | 3172165 | 3172943 | tcf7l2 (transcription factor 7 like 2) | 4.993 | 0.002 | male | yes |
| TRINITY_DN3640_c1_g1_i1 | 3721434 | 3721778 | no annotation | 4.245 | 0.007 | male | no |
| TRINITY_DN80732_c0_g1_i1 | 3723091 | 3723431 | no annotation | 4.045 | 0.024 | male | no |
| TRINITY_DN43271_c0_g2_i1 | 3724073 | 3725113 | LOC101733565 (pleckstrin homology domain-containing family S member 1-like) | 5.64 | 0 | male | yes |
| TRINITY_DN43271_c0_g1_i3 | 3725107 | 3725831 | LOC101733565 (pleckstrin homology domain-containing family S member 1-like) | 4.126 | 0.027 | male | no |
| TRINITY_DN43271_c0_g1_i1 | 3725814 | 3727694 | LOC101733565 (pleckstrin homology domain-containing family S member 1-like) | 6.344 | 0 | male | no |
| TRINITY_DN151694_c0_g1_i1 | 3742509 | 3742866 | no annotation | 5.06 | 0.001 | male | yes |
| TRINITY_DN56662_c0_g1_i1 | 3806825 | 3807132 | no annotation | 4.283 | 0.039 | male | yes |
| TRINITY_DN5848_c0_g1_i2 | 3970144 | 3989868 | vwa2 (von Willebrand factor A domain containing 2) | 9.329 | 0 | male | yes |
| TRINITY_DN452_c2_g1_i2 | 5292703 | 5293449 | shtn1 (shootin 1) | 5.254 | 0.012 | male | yes |
| TRINITY_DN3679_c0_g1_i3 | 5406344 | 5407384 | no annotation | 5.625 | 0 | male | yes |
| TRINITY_DN1115_c0_g1_i18 | 7020737 | 7051446 | pzp (pregnancy-zone protein) | -8.446 | 0.032 | female | yes |
| TRINITY_DN16274_c0_g1_i4 | 7551805 | 7776967 | multiple | 5.308 | 0.01 | male | yes |
| TRINITY_DN19099_c0_g1_i1 | 7631286 | 7631552 | LOC100487578 (alpha-2-macroglobulin-like protein 1) - intronic | 4.984 | 0.011 | male | yes |
| TRINITY_DN42788_c0_g1_i1 | 7633454 | 7633953 | multiple | 5.388 | 0.001 | male | yes |
| TRINITY_DN2504_c0_g2_i3 | 7635900 | 7885430 | multiple | 9.723 | 0 | male | yes |
| TRINITY_DN102758_c0_g1_i1 | 7651629 | 7652058 | LOC100487578 (alpha-2-macroglobulin-like protein 1) - intronic | 4.247 | 0.001 | male | no |
| TRINITY_DN101318_c0_g2_i2 | 7759551 | 7764141 | LOC100491357 (ovostatin-like) | 3.955 | 0.031 | male | no |
| TRINITY_DN1080_c0_g1_i7 | 7775636 | 7776317 | LOC108644460 (murinoglobulin-2-like) | 6.643 | 0 | male | yes |
| TRINITY_DN1080_c0_g1_i4 | 7976928 | 7994749 | phc1 (polyhomeotic homolog 1) | 10.269 | 0 | male | yes |
| TRINITY_DN1080_c0_g1_i3 | 7982811 | 8134828 | multiple | 8.553 | 0 | male | yes |
| TRINITY_DN2746_c0_g1_i10 | 7998146 | 8009403 | m6pr (mannose-6-phosphate receptor (cation dependent)) | 4.75 | 0.003 | male | no |
| TRINITY_DN20224_c0_g1_i1 | 7998819 | 7999821 | m6pr (mannose-6-phosphate receptor (cation dependent)) - intronic | 5.269 | 0.001 | male | yes |
| TRINITY_DN2746_c0_g1_i4 | 8006123 | 8009403 | m6pr (mannose-6-phosphate receptor (cation dependent)) | 6.223 | 0.001 | male | no |
| TRINITY_DN4521_c7_g1_i1 | 8133674 | 8134175 | LOC100488897 (ovostatin) | 4.958 | 0 | male | no |
| TRINITY_DN32117_c0_g1_i1 | 8524642 | 8525266 | LOC116412135 (ovostatin-like) - intronic | 4.033 | 0.02 | male | no |
| TRINITY_DN14386_c0_g4_i2 | 8664301 | 8665021 | no annotation | 3.596 | 0.015 | male | no |
| TRINITY_DN18403_c0_g1_i9 | 8686743 | 8721352 | ovos2 (alpha-2-macroglobulin like 1 pseudogene) | 4.391 | 0.001 | male | no |
| TRINITY_DN1430_c1_g2_i3 | 9452410 | 9453238 | atn1 (atrophin 1) | 9.212 | 0 | male | yes |
| TRINITY_DN26991_c0_g2_i1 | 9584961 | 9587403 | LOC116412097 (phosphatidylglycerol–prolipoprotein diacylglyceryl transferase-like) | 3.442 | 0.017 | male | no |
| TRINITY_DN20482_c0_g1_i4 | 9603480 | 9604077 | no annotation | 2.834 | 0.014 | male | no |
| TRINITY_DN5596_c0_g1_i3 | 9675146 | 9681174 | LOC100127624 (provisional ortholog of tumor protein p63 regulated 1 like) | 9.81 | 0 | male | yes |
| TRINITY_DN3866_c0_g1_i11 | 10006830 | 10008040 | gapdh (glyceraldehyde-3-phosphate dehydrogenase) | 8.856 | 0 | male | yes |
| TRINITY_DN3866_c0_g1_i10 | 10006830 | 10008040 | gapdh (glyceraldehyde-3-phosphate dehydrogenase) | 7.807 | 0.001 | male | yes |
| TRINITY_DN3866_c0_g1_i7 | 10006841 | 10008366 | gapdh (glyceraldehyde-3-phosphate dehydrogenase) | 7.166 | 0.006 | male | no |
| TRINITY_DN3866_c0_g1_i5 | 10008064 | 10008773 | gapdh (glyceraldehyde-3-phosphate dehydrogenase) | 8.072 | 0 | male | yes |
| TRINITY_DN29119_c0_g1_i1 | 10008933 | 10009139 | gapdh (glyceraldehyde-3-phosphate dehydrogenase) | 5.387 | 0 | male | no |
| TRINITY_DN328_c1_g1_i7 | 10009087 | 10011410 | gapdh (glyceraldehyde-3-phosphate dehydrogenase) | 9.381 | 0 | male | yes |
| TRINITY_DN328_c1_g1_i6 | 10010171 | 10010432 | gapdh (glyceraldehyde-3-phosphate dehydrogenase) - intronic | 6.254 | 0 | male | no |
| TRINITY_DN7512_c0_g1_i1 | 10011106 | 10011339 | gapdh (glyceraldehyde-3-phosphate dehydrogenase) - intronic | 4.052 | 0.012 | male | no |
| TRINITY_DN21880_c0_g1_i1 | 10137392 | 10137732 | ltbr (lymphotoxin beta receptor) | 6.559 | 0 | male | yes |
| TRINITY_DN12616_c0_g1_i1 | 10137920 | 10138154 | ltbr (lymphotoxin beta receptor) | 5.114 | 0.001 | male | yes |
| TRINITY_DN23111_c0_g1_i1 | 10138245 | 10138551 | ltbr (lymphotoxin beta receptor) | 6.084 | 0 | male | yes |
| TRINITY_DN2118_c0_g1_i8 | 10138785 | 10146504 | ltbr (lymphotoxin beta receptor) | 8.535 | 0 | male | yes |
| TRINITY_DN21513_c0_g1_i6 | 10147293 | 10148127 | ltbr (lymphotoxin beta receptor) | 5.152 | 0.014 | male | yes |

**Table B.** Number of transcripts that had significantly male or female biased expression in gonad/mesenephros tissue of stage 50 *X. tropicalis* tadpoles. The numbers in parenthesis correspond to the number of sex-specific transcripts – where expression was detected exclusively in male or female offspring.

| chromosome | male | female |
|---|---|---|
| Chr1 | 9 (3) | 2 (1) |
| Chr2 | 4 (2) | 1 (0) |
| Chr3 | 9 (2) | 27 (18) |
| Chr4 | 7 (2) | 4 (2) |
| Chr5 | 10 (3) | 1 (0) |
| Chr6 | 2 (2) | 2 (0) |
| Chr7 | 50 (29) | 4 (3) |
| Chr8 | 11 (1) | 3 (2) |
| Chr9 | 2 (1) | 0 (0) |
| Chr10 | 3 (1) | 0 (0) |
| scaffolds | 1 (1) | 0 (0) |
| unmapped | 3 (2) | 2 (0) |

**Table C.** Genes on chromosome 3 between 114–128 Mb in v10 genome assembly that had significantly male or female biased expression in gonad/mesenephros tissue of stage 50 tadpoles. Column names follow Table A

| TransID | Start | End | Annotation | logFC | FDR | Bias | Sex-specific |
|---|---|---|---|---|---|---|---|
| TRINITY_DN11483_c0_g1_i2 | 114682108 | 114700112 | arrdc4 (arrestin domain containing 4) | -9.265 | 0.003 | female | yes |
| TRINITY_DN4919_c0_g1_i4 | 115896740 | 115905228 | selenos (selenoprotein S) | -10.852 | 0 | female | no |
| TRINITY_DN4919_c0_g1_i2 | 115896740 | 115905228 | selenos (selenoprotein S) | -6.521 | 0.016 | female | no |
| TRINITY_DN20315_c2_g1_i2 | 116754955 | 116756417 | Xetrov90009290m.g | -8.776 | 0.004 | female | yes |
| TRINITY_DN1544_c0_g1_i4 | 117686570 | 117687358 | calml4 (calmodulin like 4) | -8.675 | 0.002 | female | yes |
| TRINITY_DN1544_c0_g1_i8 | 117686570 | 117695154 | calml4 (calmodulin like 4) | -9.076 | 0.006 | female | yes |
| TRINITY_DN1544_c0_g1_i2 | 117686570 | 117695154 | calml4 (calmodulin like 4) | -10.894 | 0.001 | female | yes |
| TRINITY_DN1544_c0_g1_i3 | 117686570 | 117701646 | overlap calml4 and cln6 | -9.332 | 0.003 | female | yes |
| TRINITY_DN4169_c0_g1_i3 | 117960573 | 117963031 | no annotation | -8.671 | 0.004 | female | yes |
| TRINITY_DN238_c0_g2_i2 | 117998782 | 117999627 | LOC108646189 (uncharacterized) - intronic | -9.242 | 0.003 | female | yes |
| TRINITY_DN2503_c0_g1_i9 | 122034576 | 122049371 | c2orf42 (chromosome 2 open reading frame 42) | 5.01 | 0.014 | male | no |
| TRINITY_DN2503_c0_g1_i12 | 122034576 | 122049371 | c2orf42 (chromosome 2 open reading frame 42) | -7.948 | 0.009 | female | no |
| TRINITY_DN826_c0_g2_i2 | 122371153 | 122374651 | gmcl1 (germ cell-less - spermatogenesis associated 1) | -9.701 | 0.005 | female | yes |
| TRINITY_DN7881_c0_g1_i2 | 123083266 | 123116702 | slc23a2 (solute carrier family 23 (ascorbic acid transporter) member 2) | -9.422 | 0.028 | female | yes |
| TRINITY_DN7881_c0_g1_i11 | 123108848 | 123116702 | slc23a2 (solute carrier family 23 (ascorbic acid transporter) member 2) | -9.75 | 0.025 | female | yes |
| TRINITY_DN2665_c0_g1_i1 | 125499506 | 125505708 | purb (urine-rich element binding protein B) | -12.417 | 0.008 | female | yes |
| TRINITY_DN2665_c0_g1_i2 | 125501145 | 125505708 | purb (urine-rich element binding protein B) | -6.722 | 0.005 | female | no |
| TRINITY_DN18664_c0_g1_i3 | 125663361 | 125664448 | vamp8 (vesicle associated membrane protein 8) | -8.133 | 0.022 | female | yes |
| TRINITY_DN1473_c0_g1_i4 | 126062381 | 126065503 | slc20a1 (solute carrier family 20 member 1) | -12.632 | 0.002 | female | yes |
| TRINITY_DN3202_c0_g1_i1 | 126356125 | 126368512 | ogdh (oxoglutarate dehydrogenase) | -10.513 | 0.017 | female | yes |
| TRINITY_DN3202_c0_g1_i14 | 126356125 | 126372002 | ogdh (oxoglutarate dehydrogenase) | -10.681 | 0.001 | female | no |
| TRINITY_DN4235_c0_g1_i7 | 127122736 | 127146974 | entpd4 (ectonucleoside triphosphate diphosphohydrolase 4) | -9.556 | 0.028 | female | no |

**Table D.** Primer pairs (in rows) used in this study. Forward and reverse sequences are given in 5' to 3' orientation.

| Locus | Forward name | Forward sequence | Reverse name | Reverse sequence |
|---|---|---|---|---|
| - | scaf835_16643_f | TATTTATTAACGCCATCATTAAAGG | scaf835_17422_r | CTTGTGGCGCAGCAGAAA |
| - | scaf7931_1217_f | GATTAAAGGCGTGGTCTGGA | scaf7931_2097_r | AACGAACGACAAAGGGAAAA |
| vwa2 | Xt_vwa2_UTR_F1 | GGAAACGGAGGCTGAAAACC | Xt_vwa2_UTR_R2 | TGTGCTGATTTTCCTGGGGC |
| bag3 | scaf8518_802_f | CCAGCAATGATATAGGGAGTCAG | scaf8518_1565_r | GGAAGGGGCTTTTGACTTTC |
| LOC108644867 | LOC108644867_f1 | GGTCCCAAGCATTCTGGATA | LOC108644867_r1 | AAGAGAGAGTGCCGCAGAAG |
| phc1 | scaf2_150071042_f | GATTGGTCCCCTTCCGTAAT | scaf2_150071793_r | GGATTTCAGGCCAGCAGTTA |
| LOC100488897 | Scaf2_f1 | TGGGCATTTGGAGGGGMATC | Scaf2_r2 | CCTGCAGCTCGACTTCTTCA |
| aicda | aicda_f1 | ATTGAATTGCTGAGCCGGTA | aicda_r1 | TTTTCTGATCGCCATTAGGG |
| LOC116406517 | trop_east_SNP1_F1 | ACAAAGCCGTGGGAGCAGTT | trop_east_SNP1_R2 | CCGGGGCTTGAGTTCCTATA |
| | trop_east_SNP1_F2 | GACGTGATGGTTCCAGATAG | trop_east_SNP1_r1 | TCCCCATTTCCTGCTTGTGG |
| LOC100127624 | trpg1l-like_f1 | GTTCTGCCTGGTTTGTGGAT | trpg1l-like_r1 | TCCCCCGGCTTAAATATACC |
| grp162 | grp162_f1 | CCCAAGGTAAACACCCCTTT | grp162_r1 | TATGGCAACCCTTTGTAGGC |
| LOC116412229* | Xetrov90017422_f1 | CCCAGCAGCTCAGCTACCTA | Xetrov90017422_r1 | TAGCCCAATCCCAATAGCAC |
| | | | Xetrov90017422_r3 | GGAATAATGCAATTACAAAT |
| LOC116412144 | LOC100493019_f1 | CCCCTTTAAGATGTGCATGG | LOC100493019_r1 | CGATGACGTTGGTTTCATGT |
| Xetrov90017432 | Xetrov90017432_f1 | ATTACATTGCCCGAATGAGC | Xetrov90017432_r1 | GATCACCGGAGCAACACTCT |
| prkg1 | trop_east_SNP4_f2 | AGAGCTGAGCATAGAAAAGCAAG | trop_east_SNP4_r1 | TCTCAAGTTGTAGCAGGCCAAG |
| TRINITY_DN3464_c0_g2_i2 | Xt_DN3464_male_F2 | CCCCACCCCTTACCCTGTAT | Xt_DN3464_male_R2 | AGGCTGTGCTCCCTCAGATA |

* LOC116412229 primers (Xetrov90017422_f1/r1) co-amplify multiple duplicated regions; for this reason, the f1/r3 primer pair were used in several individuals to amplify a male-specific allele, and confirm a sex linked SNP.

# References


1. Andrews KR, Good JM, Miller MR, Luikart G, Hohenlohe PA. Harnessing the power of RADseq for ecological and evolutionary genomics. Nature Reviews Genetics. 2016;17(2):81.

2. Glaubitz JC, Casstevens TM, Lu F, Harriman J, Elshire RJ, Sun Q, et al. TASSEL-GBS: A high capacity genotyping by sequencing analysis pipeline. PloS One. 2014;9(2):e90346.

3. Furman BL, Dang UJ, Evans BJ, Golding GB. Divergent subgenome evolution after allopolyploidization in African clawed frogs (*Xenopus*). Journal of Evolutionary Biology. 2018;31(12):1945–1958.

4. Cauret CM, Gansauge MT, Tupper AS, Furman BL, Knytl M, Song XY, et al. Developmental systems drift and the drivers of sex chromosome evolution. Molecular Biology and Evolution. 2020;37(3):799–810.

5. Mitros T, Lyons J, Session A, Jenkins J, Shu S, Kwon T, et al. A chromosome-scale genome assembly and dense genetic map for *Xenopus tropicalis*. Developmental Biology. 2019;.

6. Lahn BT, Page DC. Four evolutionary strata on the human X chromosome. Science. 1999;286(5441):964–967.

7. Wei KH, Bachtrog D. Ancestral male recombination in *Drosophila albomicans* produced geographically restricted neo-Y chromosome haplotypes varying in age and onset of decay. PLoS genetics. 2019;15(11).
