# Supplementary material for: A frog with three sex chromosomes that co-mingle together in nature: Xenopus tropicalis has a degenerate W and a Y that evolved from a Z chromosome
Source: PLoS Genet. 2020 Nov 9;16(11):e1009121. doi: 10.1371/journal.pgen.1009121 (PMC7652241; doi:10.1371/journal.pgen.1009121)
Supplement: Attachment — Submitted filename: L_to_Eds_and response_to_reviews.pdf [file pgen.1009121.s015.pdf]

McMaster University

Department of Biology
Life Sciences Building
1280 Main Street West
Hamilton, Ontario,
Canada L8S 4K1
Phone 905.525.9140
Fax 905.522.6066
http://www.mcmaster.ca

July 5, 2020

Dear Editors of *PLOS Genetics*:

We are submitting a revision of our manuscript "A frog with three sex chromosomes that co-mingle in nature: *Xenopus tropicalis* has a degenerate W- and a Y- that evolved from a Z-" for consideration by *PLOS Genetics*.

An earlier version of this manuscript was reviewed by the Associate Editor and two other experts. We found the comments to be very constructive. We have performed several new analyses that were suggested by the Associate editor and reviewers, and the results of these analyses have considerably changed some of our interpretations. Below we itemize our responses to their comments and suggestions.

Thank you for considering this revision.

Sincerely,

Ben Evans
Professor

Comments from Guest Editor:

Your manuscript has been carefully reviewed by two experts in the field, as well as by me. We all agree that these polymorphic X. tropicalis sex chromosomes present a fascinating system for the study of sex chromosome evolution and that there are some really interesting results in this paper. Nonetheless, the paper needs substantial rewriting to clarify points of confusion about the data and to tone down some of the claims about what can be inferred from these data. Furthermore, some additional analyses (detailed below in my comments and those of the reviewers) would also greatly improve the clarity and impact of the manuscript.

1. Title: "High intensity sex chromosome evolution sexualized the transcriptome of a frog (Xenopus tropicalis)"

The title needs to be changed to better reflect the findings of the paper. I agree with Reviewer 2 that it is unclear what is meant by "high intensity sex chromosome evolution". And, there is no evidence that the transcriptome of this frog has been sexualized. Indeed, there are transcripts linked to the sex determination locus that show differences in expression between males and females, but these are a very small proportion (0.05%) of the transcriptome. Furthermore, we do not know if these genes showed sex-biased expression before the evolution of these sex chromosomes or whether populations with and without the Y chromosome discovered here differ in the extent of sex-biased expression of these transcripts. Without these data, it is impossible to assign causality.

> **Response**
> Many good points are raised here (and below) and we appreciate the considerable efforts that went into the first review. In consideration of this comment and also the results of new analyses, we have changed the title to "A frog with three sex chromosomes that co-mingle in nature: *Xenopus tropicalis* has a degenerate W- and a Y- that evolved from a Z-"

2. L26-28: "These observations argue for a strong role for natural selection in sexualizing the transcriptome, with mutations in the sex-linked genomic region with sex-specific fitness effects being frequently and efficiently favored".

Again, this is an overstatement of the findings. See comment 1 about the sexualization of the transcriptome. And, the transcripts that were identified in this study as sex-biased during gonadal differentiation have not been linked to sex-specific fitness effects.

> **Response**
> We again agree with this comment and our interpretation with respect to the mechanism behind male-biased expression has changed due to the results of RNAseq analysis discussed below. We no longer speculate about natural selection as a major mechanism and instead focus on the considerable evidence we have collected that support W-chromosome

degeneration. We also no longer use the term 'sexualization' to describe our findings.

3. L39-41: “Using modern genomic approaches, we discovered rapid and intense natural selection in a small sliver of the genome that sexualized the transcriptome during gonadal differentiation.“

There are no tests for selection presented in this manuscript. Divergence between sex chromosomes can also result from neutral processes, and disentangling the roles of neutral and selective processes on sex chromosomes requires careful analyses.

Response
We agree completely, and our new analyses detailed provide support for biased expression due to nearly-neutral processes (sex chromosome degeneration) and not for natural selection.

4. L41-43: “Our results point to the possibility that three genetic variants at a single gene define the different sex chromosomes of this frog“

This is certainly a very interesting possibility, but the data presented in this paper are far from conclusive and much more work would be required before this could be determined. I think it is perfectly fine to speculate on this possibility in the Discussion but would leave such far- reaching conclusions out of the Abstract and Author Summary and instead highlight the results that you have actually found.

Response
We have removed this statement from the abstract, but retained discussion of this point in the Discussion as suggested.

5. L43-44: “These findings match theoretical predictions that many mutations have sex-specific effects on fitness“

Again, no sex-specific effects on fitness have been shown here. And it is possible that sex-specific expression of genes linked to the sex-determination locus could be due to other processes, including the possibility of different artefacts raised by the reviewers.

Response
We have removed discussion of sexual antagonism.

6. L59-61: “the extent of recombination suppression and the degree of sex chromosome divergence . . . are not necessarily positively correlated“

Certainly age and extent of recombination suppression/degree of sex chromosome divergence are not necessarily positively correlated, but I am unaware of any studies that show either a high degree of sex chromosome divergence in the absence of recombination suppression, or a low degree of sex chromosome divergence in the presence of recombination suppression. Of course, I am aware of the work in frogs in which sex chromosomes are not highly diverged, despite suppression of recombination in males.

But, rare recombination has been detected between these sex chromosomes, which is enough to account for the low levels of divergence. But, perhaps I have misunderstood the point or missed some key references. I tried to look at the review cited by the authors for this point, but could not find a clear statement in that review like the one in this manuscript.

Response
We have removed this part of the sentence.

7. L67-68: “wherein the function (i.e. whether female or male determining)“

This is confusing as stated here, because changes in heterogamety are discussed later in the paragraph, and because transitions can involve male-determining to male-determining factors (or female-determining to female-determining). I think here it might be more clear to say “wherein the identity or the genomic location of the sex determining locus changes”.

Response
We have clarified this point as follows ‘Another phenomenon that may influence recombination and divergence between sex chromosomes is turnover, wherein the genomic location, genetic function (i.e., whether female or male determining) or gene that triggers sexual differentiation changes‘. We choose this wording because it accommodates the possibility that the same gene might trigger male differentiation in one species but female differentiation in another.

8. L76: perhaps also cite papers in salmonids for translocation of an existing sex determination gene; e.g. Yano et al. (2013) Evolutionary Applications 6: 486-496.

Response
We agree and have added this citation.

9. L131-133: I wasn’t sure why the YY individuals were mentioned here until reading further. I agree with Reviewer 1 that it might be nice to make a figure with the possible crosses and sexes of the different chromosome combinations. I drew one for myself!

Response
We agree and have added this as a new Fig. 1 in this revision.

10. L129-143: Within this paragraph alone, the different sex chromosome genotypes are referred to as ZW or WZ, YZ or ZY, and YW and WY! It is already challenging for the reader to follow these different combinations; please use one order for each genotype combination and then apply it consistently throughout the paper.

Response
We have editing the manuscript to always have the W-chromosome first and the Y-chromosome last in all sex chromosome genotypes.

11. L144: Like Reviewer 2, I also stumbled over the point that three crosses are introduced earlier but only two are mentioned here. Perhaps in the very helpful overall introduction to the data (lines 120-127), this could be clarified.

Response
We have clarified the use of the three families in the text and also on the right side of Fig. 1.

12. L161-162: the statement: "and that at least one (and possibly both) of the parents did not carry a Z chromosome" needs more explanation. I am guessing that it is because the numbers are too small in the crosses to be confident that the sex ratios are really 1:1?

Response
We have clarified this point by referring to the new Fig. 1 and clarifying text that refers to this inference in several places.

13. L174-197: I find this section confusing as well as troubling that the region of sex-linked markers differ between the two families. I appreciate that you did extensive Sanger sequencing to try and identify additional, shared markers that were sex-linked across the populations and in the two crosses. This was a lot of work and did not yield very conclusive results. It is not necessary to do so for this paper, but one option that has worked very well for my lab is to determine whether any of the SNPs identified in your RAD-seq data are in restriction sites. You can then design PCR primers flanking these SNPs and simply do a restriction digest of the PCR product and run the products on an agarose gel to diagnose whether an individual is homozygous for the presence of the allele that creates a cut site, heterozygous, or homozygous for the allele that does not contain the restriction site. Alternatively, you could scan the genomic sequence from this region for microsatellites, and design primers flanking those microsatellites, to see if any show patterns of sex-linkage. This approach has also worked well in my lab for identifying recombination boundaries on sex chromosomes.

Response
We agree that it would have been more straightforward to interpret the results from each family if they had a shared completely sex-linked SNP. We have clarified in the text several points that account for these differences. First differences in coverage and polymorphism in restriction enzyme sites can cause drop out of RRGS sequences in each family. Second, natural variation in recombination in different individuals is expected to cause the margins of sex-linkage to vary. And third, we suspect that the sex chromosome genotypes of the parents of Family 1 and 2 may differ, and that this could account for differences in sex linkage of the distal (telomeric) region of the sex-linked region. Specifically we speculate that the sex chromosome genotype of the father of Family 1 was ZY, whereas that of the father of Family 2 is now demonstrated to be WY. This is explained in detail in this revision.

For the paper, I think it could be more clearly explained that the Ghana East family shows that there is no recombination with SNPs between 2.6 and 6.5 Mb and sex phenotype: the next marker is somewhere after 10Mb, suggesting that the male-determining factor is within the first 10Mb of the chromosome; you just don't have any markers to say where the boundary is. The marker most strongly associated in the Ghana West family suggests that the male determination factor is between 8.1 and 13.79, with the peak at 9.149. So, these intervals do overlap, but the data are shaky.

Response
We acknowledge this point with this new sentence: "In the Ghana East family we lacked markers between ∼6-11Mb on chr7, and consequently were unable to further pin down the medial extent of sex-linkage in this family using the RRGS data."

One idea would be to look for sex-linked SNPs in the RNA-seq data of the third cross, which also was derived from the same Ghana East father as the mapping cross and see if additional resolution of the sex-linked region can be found. This would hopefully increase confidence that the sex-determination locus is indeed the same in these two populations/crosses.

Response
What a fantastic suggestion. We did this and the results were very illuminating (thank you!). We have a new figure (Fig. 5) and supplemental figure (Suppl. Fig. S6) and important additions to the Results and Discussion that are also now highlighted in the title and abstract. I honestly am still amazed at how clean these results turned out to be (!). We were lucky that the sex chromosome genotypes of Family 3 ended up being a complicated one (a WZ female x a WY male) which permitted many conclusions. We did not know these genotypes in advance, and this particular type of cross made possible several exciting findings.

Analysis of within individual RNAseq polymorphism of expressed sex-linked transcripts allowed us to pin down the sex chromosome genotypes of both parents of Families 2 and 3, and show that the W-, Z-, and Y-chromosomes were all collected in individuals from the same small seep in Ghana. We also used this information to go a step further in this revision by performing differential expression analyses on subsets of offspring with different putative sex-chromosome genotypes. This is discussed in the Results and Discussion and several new supplemental figures are included that show the results of these analyses (Suppl. Figs. 7-9). All of these conclusions are newly presented in this extensively revised revision.

14. L109-110: I agree with the Reviewers that these tests would likely be much more powerful if split between the different populations!

Response
I think the line numbers being referred to here are L209-210, where we discussed the Fst results in the previous submission. We have explained in the text the rationale for focusing on comparisons between the sexes across populations rather than comparisons within populations, i.e., because we do not know the sex chromosome genotypes of almost all individuals in the RRGS data.

15. L208-220: This paragraph is very confusing. I think it would be much clearer for the reader if you first explain that there are differences in the order of the assembly v9 and v10 in this region of the genome, and that you are trying to compare the locations of markers identified as sex-linked in a ZW system with those identified here.

Response
We have clarified this point as follows "There are differences in the v9 genome assembly used by Mitros et al (2019) and the v10 genome assembly used in this study in the sex-linked region of chromosome 7 (Suppl. Fig. S3). To facilitate comparison between these studies, we report genomic coordinates of both assemblies for the Fst and Sanger sequencing results; other findings are reported using v10 coordinates."

16. L237: "although we did not assess sex-linkage in this cross". You certainly could do this with the RNA-seq data, using a method like SEX-Detector (Muyle et al. (2016) Genome Biology and Evolution 8: 2530-2543), which would provide additional markers and increase confidence in the concordance of the position of the sex determination locus in the two different populations from Ghana.

Response
We looked into this approach and unfortunately it requires RNAseq data from the parents and offspring. Here, we only have RNAseq from offspring because we were unable to obtain the corresponding tadpole tissue from the field collected adults. However, as detailed above and below, we have now added new analyses that leverage our RNAseq data to quantify the extent of degeneration of the W-chromosome.

17. L241-244: This sentence is not particularly clear. Also you found 151 of 259,197 mapped transcripts were sex-biased (0.05% of transcripts). The clustering of these transcripts is definitely interesting, but I do not see that this is the "sexualized transriptome" referred to in the title and abstract.

Response
We agree and have removed this term throughout.

18. L248-249: "suggestive of stronger sexual selection in males". This is an overstatement. Is a difference of 62 male-biased vs 43 female-biased transcripts of 259,197 total transcripts significant? And, what does expression during gonadal development have to do with sexual selection?

Response
We have removed discussion of sexual selection.

19. L267-286: I find it over-reaching to immediately jump to the sexual antagonism explanation for this result. First, you need to consider the possibility that there are artefacts that could explain this result. The possibility of mapping errors due to using a female genome without the Y chromosome absolutely needs to be considered (suggested by Reviewer 1) as does the possibility that the ancestral W is degenerated in this region and therefore the reduced expression in females simply results from missing sequence from the W chromosome (suggested by Reviewer 2). It is not clear whether the genome assembly was generated from a ZW or WW female; if a ZW female, it is possible that the assembly represents mostly the Z chromosome if the W has experienced degeneration. You might be able to assess this by examining your RAD-seq data: if all of the males in your crosses carry a degenerate W chromosome, then you would find reduced number of reads (relative to the genome-wide average) in your RAD-seq data in the sex-linked regions.

Response
Unfortunately our RADseq data were not useful for this purpose in part because there is high variation in coverage among individuals but more importantly because we do not know the sex chromosome genotypes of most individuals for which we have RADseq data. However, the RNAseq data allowed us to overcome this issue because we were able to resolve individual sex chromosomme genotypes, pool individuals with the same genotype, and perform new differential expression analyses on specific subsets of individuals.

If the results are not due to an artefact, then why would metabolic genes like mannose-6-phosphate receptor (accounting for 3 of the sex-biased transcripts) or gapdh (accounting for 8 of the sex-biased transcripts) be under sexually antagonistic selection during gonadal development? Furthermore, I don't really understand the argument about why it is useful to compare the expression level of the sex-biased genes to the expression level of non sex-linked, non sex-biased genes? This argument needs to be clarified.

Response
This section has been removed.

20. L307-309: It would be really interesting to present more detailed data on the male and female meiotic events in these two families in this region of chromosome 7. A supplementary table or figure would be fine. Is there any evidence for suppression of recombination in male meiosis in this region of the genome?

Response
We have added text that argues that there probably is recombination suppression between the W- and Y-chromosomes but not between the Z-

and Y-chromosomes. This is based on a combination of inferences from the RRGS and RNAseq data.

21. L346-348: If your hypothesis is correct that there are three different variants at the sex determination locus, then you would expect it to be present on all the sex chromosomes. In this case, it would be interesting to know whether any of the genes that are more highly expressed in developing male gonads are interesting candidate genes for the sex-determination locus? With the RNA-seq data, you should be able to look for sex-specific/sex-linked SNPs in these genes that might lead you to a good candidate gene!

Response
We agree and this was the initial motivation for selecting this particular tissue type and developmental stage. In the initial submission and this revision, Table S2 lists sex-linked transcripts that are differentially expressed. Unfortunately none of these have annotations that seem particularly sex-related and some are not annotated at all. There is also a similar table of annotated genes in this region in the paper by Mitros et al. 2019

22. L401-402: "pronounced nucleotide divergence". I am not sure that an Fst of 0.05 counts as "pronounced".

Response
We have changed "pronounced" to "more substantial"

23. L465-467: Why is this surprising? If sexually antagonistic selection is driving these patterns, as you claim, the pseudoautosomal region is predicted to be an excellent place for sexually antagonistic mutations (See Otto et al. (2011) Trends in Genetics 27: 358-367; Jordan and Charlesworth (2012) Evolution 66: 505-512; Charlesworth et al. (2014) Evolution 68: 1339-1350).

Response
We have removed this portion of the discussion.

24. L468-483: Here you should probably cite a recent review from Sardell and Kirkpatrick (2020) American Naturalist 195: 361-379, which provides a comprehensive overview of the generality of these sex-specific recombination patterns and possible explanations. Another nice and interesting review is Brandvain and Coop (2012) Genetics 190: 709-723.

Response
Thank you; we added these citations.

25. L514-515 "It is fascinating that these pronounced sex-specific fitness differences can emerge so early in development – at or before the earliest steps in primary gonadal differentiation."

Again, please be clear about what you have shown, which is sex-biased gene expression, not sex-specific fitness differences.

Response
We have removed this sentence.

I know this is a lot of comments. I hope these suggestions are useful for you; they are certainly intended to be constructive and help you improve your manuscript!

Response
We sincerely appreciate your thoughts and time; these comments were **extremely** helpful; thank you!

Reviewer's Responses to Questions

Comments to the Authors: Please note here if the review is uploaded as an attachment.

Reviewer 1: I found this manuscript very hard to follow, and below I comment on some text that is too detailed to be understandable, or too long-winded. Line 116 states the main goals as studying X. tropicalis to

(i) test for male or female heterogamy in natural populations (ii) narrow down the sex-linked region (iii) study sex-biased expression [and nucleotide differentiation, which probably belongs under point ii] (iv) characterize patterns of recombination in both sexes of wild-caught individuals of this species

The study seems to have detected a region that appears to suggest male heterogamety, whereas some previous work suggested female heterogamety. It does identify a sex-linked region, though it was not clear enough whether this is the same region in both these systems (the abstract suggests this, but the reasoning is not clearly explained). I have some reservations about the conclusions under aims (iii) and (iv).

It is no longer surprising or astonishing (lines 18 and 60) that genetic sex-determining systems differ (it's not clear what 'extensively' means) among – and even within – species. This has been known for many decades, with many well-studied examples, and line 90 cites three other cases in frogs, -— the question is whether the frog system studied can add new understanding.

Response
We agree and have removed these modifiers.

Line 83 says, correctly, that 'Understanding the drivers of [or, better, the selective forces causing] sex chromosome turnovers is challenging (reviewed in [18]), but catching them in the act – during evolutionary windows where multiple sex determination systems co-exist in one species – may help us understand why and how they occur'. Yes, studies should be done, but please tell us here precisely what the questions are.

Response

We have clarified this point as follows "Specifically, these transitions may offer insights into whether and how characteristics of ancestral sex chromosomes (e.g., nucleotide divergence, sex-biased expression, degeneracy) bleed through to effect new sex chromosome systems."

It is also confusing to write 'three sex chromosomes'. Even the term 'sex chromosomes' (and Y chromosome) may be misleading in the system studied, as these are sex-determining genes with none of the distinctive characteristics of sex chromosomes. If these terms are used, it needs to be explained that they are used purely for brevity, but that the situation is very different from that of more familiar sex chromosomes whose Y chromosomes include non-recombining regions.

Response
We have clarified this as follows " Most of the sex chromosomes of *X. tropicalis* are pseudoautosomal regions where genetic recombination occurs (Bewick et al. 2013)."

The most interesting finding, which IS novel, is that a small 'sex-associated' part of the sex chromosome pair in this frog can be detected by higher sequence 'differentiation', but is not completely sex-linked, yet the region has a 50-fold enrichment of transcripts with male-biased expression during early gonadal differentiation, compared to the rest of the genome. The information about sex-differences in the rates and genomic locations of recombination is also valuable.

Response
We now have an alternative interpretation where the sex chr genotype of the parents provides explanations for the extent of recombination and differences between the families. These results have led to a more nuanced interpretation that recombination is probably suppressed in the sex-linked region of the W-chromosome and the Y-chromosome, but not between the Z-chromosome and the Y-chromosome.

DETAILED COMMENTS

In line 198, the heading has a typo, and should read 'The X. tropicalis Sex Chromosome Has a Small Region of Differentiation Between the Sexes'.

Response
Corrected

Fst between the sexes can identify the sex-linked region much more precisely than genetic mapping in families (with limited progeny numbers, see comment below), and can also tell one which sex is heterogametic for the region. The ms states that all of the females have a W-chromosome, but does not explain how this is known, and whether it is consistent with the genetic results from Ghana frogs. ['differences in nucleotide polymorphism ... Is' should be corrected to be in the plural, though the sentence can be omitted entirely, and indeed most of the paragraph]. Something called v10 is mentioned,

but not explained.

> **Response**
> We agree. We have corrected the grammar to be plural and we have defined v9 and v10 (which refer to different assembly versions) at first use. In response to a similar concern by the Associate Editor, we have elaborated on some key differences between these assemblies in the Results section just before we discuss Fst.

The paragraph starting in line 208 tells us that a peak in Fst occurs at the genome assembly location where a W factor was previously inferred. This text could be made shorter and clearer by simply referring to Figure 2 (and S2), together with mentioning which population it refers to, and the paper(s) with the previous genetic result. I was left unclear whether populations with male and female heterogamety all have an Fst peak in the same region, as Figure 2 seems to combine several population samples. It is also not clear what genome-wide Fst values are seen between the sexes in the sampled individuals.

> **Response**
> One of the new results in this revision that was surprising to us is that the W-, Z-, and Y-chromosomes were all detected in the population from east Ghana and probably also the population from west Ghana. Given these results, we feel that the most sensible analysis is to pool individuals based on an important and unambiguous datum: the phenotypic sex of each individual.

To understand the gene expression results, readers need to understand the extent of the region that has high Fst between the Y and X, or W and Z, depending on which system is being analysed. Figure 3 appears to show that at least 20 genes within a few Mb have high M/F ratios. It would be helpful to tell us the actual size of the region, and the raw fold difference represented by the y axis, so that we can know if these are all large differences (rather than just the log values, where differences are harder to see).

> **Response**
> In the discussion on Fst results we provide coordinates of the Fst peak. However, we are unable to say specifically whether this corresponds to a male or female heterogametic system because we do not know the sex chromosome genotypes of almost all individuals in the RRGS dataset (the two parents of Family 2 are the only individuals in the RRGS data for which we were able to resolve the sex chromosome genotype). We have clarified this as follows "Because we do not know the sex chromosome genotype of almost all individuals for which we performed RRGS (the parents of Family 2 are an exception, see below), we were unable to evaluate between cohorts of females and males that each had the same sex genotype."

For the genes with differential expression, the coordinates of the start position of each transcript are provided in Supplementary Table S2 (for v9 and v10) and are graphically illustrated in Fig. 4 (previously Fig. 3). Transcripts with differential expression extend distally (toward the telomere) from the Fst peak. We have edited the legend of this figure (which was formerly Fig. 3) to clarify that the y-axis uses the standard $\log_2$ transformation to quantify the male/female transcript expression ratio. Thus a y-axis value of 1 corresponds to a two-fold higher expression level in males compared to females, whereas a value of 5 corresponds to a 32 fold higher expression level in males compared to females.

It should be made clear whether this is a 'normal' gene density for this genome (or for a chromosome end), or out of line in either direction, and whether the GC content could be affecting the expression results (I do not know if this is a potential problem, but it is known to affect DNA sequence coverage estimates).

Response
We present genome-wide $\log_2$ male/female expression ratios for individual transcripts in Suppl. Fig. S5. From this figure, and also the closeup of chr7 in Fig. 4, it is clear that the distribution of genes is relatively uniform and not especially concentrated on the end of chr7. Although we did not specifically quantify GC content, the expression analyses we performed use a k-mer based approach that does not require mapping of reads to the assembly. Thus we do not think this variable has an impact on the expression analyses. For conclusions based on transcripts mapped to a genome assembly, or reads mapped to our transcriptome assembly, it is conceivable that GC content has a some effect on mapping efficiency. We have clarified this point in the Supplement as follows "As discussed above, other factors such as GC content and repetitive sequences also may have contributed to mapping error of transcripts to the genome assembly or of RNAseq reads to the transcriptome assembly. However, we have no reason to suspect GC content would disproportionately affect mapping in certain genomic regions (e.g., sex-linked and non-sex-linked) or in a specific sex."

The gene expression results should also be evaluated carefully, and any other caveats should be discussed. A recent study found that sex differences were due to 'reference bias' in a species where the reference genome was a female in an XY species, and new work (by the same lab) that used reference genomes from the males used to estimate expression did not detect the expression differences that had been claimed previously (ZHOU AND BACHTROG 2012; WEI AND BACHTROG 2019). This possibility should be excluded in future work, including this study (where, of course, it may not be a concern, but, if so, readers should have it explained why not). I therefore did not review the section about expression in detail. If this concern is not justified, it should be explained why it can be excluded.

Response
We agree that this is an important concern. The incorrect inference of downregulation of Y-linked alleles in heterogametic (XY males identified by Wei and Bachtrog 2019 initially involved use of a homogametic (XX) female reference sequence by Zhou and Bachtrog 2012. In this study, we assessed expression relative to a *de novo* transcriptome assembly. Reference bias could have affected mapping of male-specific transcripts to the female-genome, but this should cause an inference of lower, not higher, expression in males. We also include a brief discussion of an analysis of unmapped transcripts. We have added a discussion of this issue to a new section in the Supplement ("Reference Bias"

In addition, the use of the gonad/mesonephros complex during an early stage of sexual differentiation should be justified. Clearly, such tissue is relevant to finding the sex-determining gene(s) but it is not clear to what extent other genes expressed only in gonad tissue will be included.

Response
We have added context to the Methods section as follows: "Although this study does not explore this issue, the initial motivation for selecting this tissue and developmental stage was that it corresponds with the timing of gonadal differentiation in *X. laevis* (Yoshimoto et al. 2008) and the sex determining gene of *X. tropicalis* could also be expressed in this tissue type and developmental stage. "

There seems to be no heading for the section about recombination (line 296 onwards), and the text does not actually describe mapping results, or the marker numbers, or really explain the reasoning for any conclusion. I was not sure whether linkage groups were based on enough markers to be confident that markers near the ends were included. The text says 'There was a strong relationship between map length and chromosomal coverage in females map' [meaning the female map of an unspecified family], but here 'coverage' seems to mean something that is not defined, and Fig. 4, which is stated to show the result, doesn't use it. I think that the authors are trying to explain that total genetic map lengths in females increased with the chromosomes' lengths [measured in an unspecified way, maybe the assembly lengths?], but in males were unrelated to these lengths, which suggests that crossover events in males are concentrated in limited regions. However, it is unclear whether the genetic and physical — assembly — maps are good enough to make this convincing.

Response
The heading of this section is "Rates and Locations of Recombination are Sex-specific in *X. tropicalis*". In the first paragraph of this section we enumerate the number of markers (SNPs) that were used in the analysis for each family.

We apologize for the confusing wording we used in the first submis-

sion. We now refer to comparisons of linkage map length to "the size in base pairs of the genomic region to which the linkage map corresponds" and reserve the term "coverage" to the standard usage of this term to refer to the sequencing depth (in this case of the RRGS data).

Given these caveats, and the need for extensive shortenings and clarifications, I did not review the Discussion section.

MINOR COMMENTS

[Please note that the line numbers are exceedingly small, and sometimes I may not have the correct one, as I could not always read them. The text is also small and very difficult to read on a laptop]

Response
We apologize! The font of the line numbers is now larger.

A general comment is that the text is long-winded and could be shorter and clearer. For example 'A remarkably high number of changes amphibian sex chromosome systems is evidenced by variation among species in whether males or females are the heterogametic sex'. Means simply 'In amphibia, many cases of changes between male and female heterogamety have been inferred [or discovered]'.

Response
We have changed this text to be similar to this suggestion. More generally we have thoroughly edited the manuscript for clarity and streamlined the Discussion by deleting several sections. The Results section is longer because we performed new analyses that were suggested by the Associated Editor.

Line 100 onwards could also be shortened and made clearer. The important point is that the ancestral sex determining system of subgenus Silurana, which includes X. tropicalis, was inferred to have female heterogamy, implying that the X. tropicalis Y is derived either from the W-chromosome or the sex chromosome that is shared with other species, which is referred to as the Z-chromosome [28].

Response
We have slightly shortened this section.

Line 51: expropriated does not seem the right word

Response
We agree and have removed this wording.

Line 61: It is confusing to say that differences in recombination suppression do not correlate with the degree of sex chromosome divergence, as divergence will largely occur only once recombination is suppressed.

Response

We have removed this statement.

It is also not clear enough to say that ‘dosage imbalances‘ are a problem, as the main problem when Y or W chromosomes lose genes is that the amount of gene product in the affected sex may be lower than optimal, which is not what readers will understand from this phrase (they will think of the cases where balance is needed between the expression of two or more genes in a multi-protein complex, which may contribute to the evolution of sex chromosome dosage compensation, but are very rare).

Response
We have clarified this point as follows “The ability to cope with differences between the sexes in the dosage of gene products stemming from degeneration of sex-linked alleles on the W- or the Y- chromosome (Adolfsson and Ellegren, 2013), periodic recombination (Perrin 2009), and genomic conflict associated with mutations with sexually antagonistic fitness effects via the origin of sex-biased expression patterns (Vicoso, 2013) all may influence whether or not – and how much – sex chromosomes diverge from each other.”

These problems can be corrected by shortening the Introduction to focus on the points relevant to this study, including the very long-winded description of turnover events, which can be briefly outlined, with a reference to a recent review.

Response
We have removed or combined several portions of the Introduction in response to this concern.

Line 81: Is heterogamy the correct word?

Response
We have changed this to read “Consequently, when a turnover event does not involve a change in which sex is heterogametic, it may be difficult to distinguish between translocation of an ancestral sex determining locus and neofunctionalization of a novel locus.”

Line 92: ‘segregating male-linked and female-linked variation‘ is not correct terminology. It should be something like ‘variants that segregate in families as Y- or W-linked‘, and ‘no cytological divergence among sex chromosomes of this or any other Xenopus species has been detected‘, means ‘no Xenopus species with genetic sex-determination has heteromorphic sex chromosomes‘.

Response
We have removed the first text but retained the use of the ‘cytological divergence’ in order to make clear that we are referring to heteromorphic sex chromosomes that can be identified microscopically and that we are not referring to diverged sex chromosomes that may be distinguished by structural changes (nucleotide divergence, insertion/deletions) that are

not cytologically visible.

Line 130: ‘dominant for female differentiation over the Z-‘ is not well expressed. Maybe change to something like ‘Relative to the Z, defined above, the factor carried by the ‘W‘ dominantly determines femaleness and the ‘Y‘ factor is male-determining‘. The following text is hard to understand and can be cleaned up and shortened (it repeats things unnecessarily).

Response
We have clarified this section in the Introduction and also reduced some of the redundant verbiage from the Results section.

Maybe a Supplementary figure can show the expected progeny ratios of the crosses, and be referred to to state the conclusion clearly and briefly, for example, as follows (some comments are in []) ‘Two small families generated from individuals from the western and eastern edges of Ghana, provided unambiguous evidence for male heterogamety. The sex ratio was close to 1:1 in both crosses (22 females and 21 males or Ghana west, and seven females and five males for Ghana east). Several paternal SNPs at the distal end of chromosome 7 were associated with sex, and several SNPs were completely Y-linked in each family (though the sex-linked SNPs differed in the two families), but no heterozygous SNPs in the maternal frogs were identified as sex linked in either family (Fig. 1). Thus, at least one (and possibly both) of the parents in both crosses did not carry a Z-chromosome [here, I was not sure why this is inferred — should it be W chromosome?].

Response
We agree and have added a new Fig. 1 that illustrates the expected sex chromosome genotypes that can be created by different parental sex chromosome genotypes

After FDR correction, five SNPs in the Ghana west sire were significantly associated with offspring sex [I am not sure why genome-wide ‘heterozygosity‘, which is not defined, is mentioned here, as the significance test is surely all that is needed], and three in the Ghana east family.

Response
We have added this statement to clarify why we report heterozygosity in each parent: “..., which suggests that the power to detect female-linked and male-linked variants is similar”

[It is important to understand that complete sex linkage in such small families does not mean that no recombination occurs in the region studied — to detect complete sex-linkage, one needs to use an adequate natural population sample.]

Response
We completely agree and did not mean to suggest otherwise; please see next response.

Some non-sex-linked SNPs in the Ghana west family also varied in the Ghana east family, and were sex linked, and vice-versa, probably reflecting the small family sizes, such that recombination events may not occur in a given family; overall, the results identify a sex-linked region, and not a non-recombining region.

Response
Based on the results of new analyses, we infer that the sex chromosome genotype of the parents of Family 1 and 2 was different and that differences in recombination suppression between these genotypes is a likely explanation for the differences in the extent of sex-linkage in these crosses.

In small samples of wild caught individuals, Sanger sequencing identified at least one fully sex-linked SNP in the Ghana east Ghana east population, but it was not fully sex-linked in the Ghana west one, or in either family (Table 1). [Again, the text can be greatly shortened and much more clearly written].

Response
This section has been modified for clarity.

References Wei, K. H.-C., and D. Bachtrog, 2019 Ancestral male recombination in Drosophila albomicans produced geographically restricted neo-Y chromosome haplotypes varying in age and onset of decay. PLoS Genetics 15: e1008502. doi: 10.1371/journal.pgen.1008502 Zhou, Q., and D. Bachtrog, 2012 Chromosome-wide gene silencing initiates Y degeneration in Drosophila. Current Biology 22: 522–525. doi: 10.1016/j.cub.2012.01.057

Reviewer 2:

X. tropicalis is a great system for studying sex chromosome turnover, as it has polymorphic ZW and "WY" sex-determining systems (derived from an ancestral ZW). In this manuscript, Furman et al use a combination of genetic and sequencing approaches to characterize the sex-linked regions of ZW and YW individuals. I really enjoyed the manuscript, but I struggled to interpret some of the data, to a large extent because not much information is provided/available on the ancestral pair of sex chromosomes. Specifically:

- An important part of the puzzle is how differentiated the ancestral ZW pair is, and whether the Y chromosome is derived from the Z or the W. I think this could really change the interpretation of the differentiation and gene expression patterns. For instance, if the W-specific region has reduced expression due to degeneration, and the Y is derived from the non-degenerated Z, then WW females may have lower expression than WY(Z) males. The fact that a similar excess of male-biased genes was found in ZW X. tropicalis (ref. 52) is consistent with this scenario.

Response
We completely agree and (excitingly) new analyses in this revision demon-

strate using the RNAseq data that the Y-chromosome is derived from the ancestral Z-chromosome (please see Fig. 5 and associated discussion).

- More information is in places needed to make sense of the results. For instance, it would be very helpful to state early on what is known about the sex chromosomes of the lab strains used here, and if at all inferred, those of the Sierra Leone and Nigeria individuals. If this is not known, then it should be explicitly mentioned as an important caveat.

Response
In this revision we have stated that we do not know the sex chromosome genotypes of almost all individuals in the RRGS data as follows: "Because we do not know the sex chromosome genotype of almost all individuals for which we performed RRGS (the parents of Family 2 are an exception, see below), we were unable to evaluate between cohorts of females and males that each had the same sex genotype."

- FST analysis: I found it confusing that all genotypes were combined; which sex chromosome is differentiated, if we don't know the genotypes of the individuals used for the Fst analysis? Would it be possible to plot separately the differentiation between: females(ZW) and males (ZZ) females (WW) and males (WY)? Or at the very least to plot this for only the WW/WY individuals?

Response
We agree that this would be wonderful to do with the RRGS data but, as now further clarified in the Results we do not know the sex chromosome genotypes of almost all individuals that were used for the RRGS analysis. The good news is that new analyses of RNAseq data did provide this information for offspring of family 3, and we have performed analyses of differential expression between subsets of individuals with the same putative sex chromosome genotypes (Suppl. Figs. S7, S8, S9). As this reviewer suspected, knowing this information has a huge effect on the inferences we were able to make and led us to the conclusion that the W-chromosome is degenerate and that the Y-chromosome evolved from an ancestral Z-chromosome.

- Sanger sequencing: I apologize if I missed it, but I could not find how these loci were chosen for sequencing. Are they just all the loci that had a sex-linked SNP in the crosses? A few sentences in the methods and results would be very helpful even if the details are in a supplementary file.

Response
We targeted regions that were more or less evenly spaced throughout the sex-linked region identified by Mitros et al., and also towards the telomeric region. We have clarified this as follows "A focused survey of sex linkage was also performed using Sanger sequencing of amplicons from

different portions of the sex-linked region.” As this reviewer suspected, we initially directed our primer design towards SNPs in the RRGS data, but because the amplifications were very patchy, we turned to other regions, so the connection to the RRGS data became tenuous, and this was not mentioned.

- P5L120: "generated three families" – I could not find information about the third family. Is this a typo? If not, how many individuals were sequenced? Did you find evidence of a ZW system there?

Response
We agree this was unclear and this concern was shared by the Associate Editor and the other reviewer. We have clarified the three families used in this study in Fig. 1.

- P7L184: "we were able to identify at least one 100% sex-linked SNP in the Ghana east laboratory cross and the Ghana east wild population" – This makes it sound as if the same SNP was fully sex-linked in the crosses and in the wild population, which does not seem to be the case if I understand Table 1 (i.e. no locus is classified as “Y“ in both columns).

Response
We have clarified this point as follows “Using Sanger data, we were able to identify one 100% sex-linked SNP in the Ghana east laboratory cross and (with a very small sample size) three 100% sex-linked SNPs in the Ghana east wild population, but none in the Ghana west laboratory cross or the Ghana west wild population.”

It also seems that only a single female was sanger-sequenced for the wild Ghana east population (and only 1 to 3 males for these "Y" loci), so it seems a bit optimistic to say that you found a 100% sex-linked SNP in this population?

Response
We completely agree and include this caveat in the text above “(with a very small sample size)”

- I found the title confusing. What is high intensity evolution, and what evidence of it do you find? What would low intensity evolution of a sex chromosome look like?

Response
We agree and the title has changed

Other:

P5L114: I think quite a bit is known about when sex chromosomes become differentiated, and maybe a couple of citations would make sense here.

Response

We agree and have cited a review paper by Bachtrog (2013) here.

P5L131: “YY individuals can only be generated if“ –> from the rest of the sentence it sounds like they can in fact not be generated? I was a bit confused by this.

Response
We have clarified this text.

Comparing the expression of the sex-biased genes to the average expression in the sample does not seem very reliable. A better approach would be to use X. laevis gene expression as a proxy for ancestral expression to check if there has been up- or down-regulation (e.g. https://www.ncbi.nlm.nih.gov/Traces/study/?acc=SRP167133). But this is merely a suggestion as it is not necessarily within the scope of the present manuscript.

Response
We have removed this analysis from the paper.

Similarly, it seems a shame not to have taken advantage of the SNP information in the RNA-seq data, especially given that the RAD-seq data seems to have somewhat low/inconsistent coverage.

Response
We did do this in this revision. As itemized above and in the manuscript, the results from this analysis really pushed forward our understanding. We are very grateful for this suggestion; thank you!
