# Supplementary material for: A frog with three sex chromosomes that co-mingle together in nature: Xenopus tropicalis has a degenerate W and a Y that evolved from a Z chromosome
Source: PLoS Genet. 2020 Nov 9;16(11):e1009121. doi: 10.1371/journal.pgen.1009121 (PMC7652241; doi:10.1371/journal.pgen.1009121)
Supplement: Attachment — Submitted filename: Responses_2.pdf [file pgen.1009121.s016.pdf]

Comments from Guest Editor:

Both reviewers and I agree that the manuscript is improved with the new analyses. The results are quite interesting in that there is evidence that a new Y chromosome has evolved from an ancestral Z chromosome. Of course, confirming this hypothesis will take a much more detailed molecular analyses than that presented here, but this study lays the groundwork for these longer-term analyses. The main challenge is that the current manuscript is a difficult read that makes the reader work extremely hard to understand what has been found and the significance of the findings. It feels like the results have not yet been fully digested by the authors of the paper and that they can no longer "see the forest for the trees". Reviewer 2 points out that the manuscript is still written in a historical manner and suggests some re-organisation that might help. I agree with this suggestion, as I detail below. My extensive (although not comprehensive) comments are aimed at helping the authors present their results more clearly and concisely. In addition, and as noted by Reviewer 1, I also found many small typos and grammatical errors throughout the manuscript. Not all have been indicated, and it would probably be useful for someone not associated with the study to give this manuscript a very careful read before it is resubmitted.

I am happy to re-consider another version of this manuscript, if the authors are willing to significantly revise in order to make it clearer and more accessible.

Overall organisation

1. I agree with Reviewer 2 that it would help the reader a lot to present some of the results of the analyses of the RNA-seq data of Family 3 in the first part of the paper. Although there is no RNA-seq data from the parents of this cross, the analyses of nucleotide diversity in these data is extremely illuminating for inferring the sex chromosome status of the parents of both Families 2 and 3. As such, this should be presented sooner. Essentially, I would move the text from L311-344 to the first section of the Results, after presenting the mapping results for Families 1 and 2.

> **Response**
> We agree and have moved this portion of the RNAseq analysis forward in the Results to the section "Evidence of Genetic Degeneration of the Sex-linked Region of *X. tropicalis*, and that the Y chromosome is derived from an ancestral Z chromosome"

The results in L345-374 seem a little tangential could be shortened and/or moved to the Supplement (see more detailed comments below).

> **Response**
> We have moved this section to the supplement

An even more radical rewrite might involve presenting the nucleotide diversity analyses from the RNA-seq analyses of Family 3 first, and then present the mapping results of Families 1 and 2. This could helpful because it would provide the probable

sex chromosome status of the parents, at least of Family 2. But it is hard to know which is better without trying.

Still even more radical would be to reorder the entire results section. I would start with the population structure analyses. This would provide the reader with a better context for the populations studied here. Then, the Fst analyses could come next, as it identifies a possible sex-linked region in this broader set of populations and shows that it is the same genomic region as the previously identified W-chromosome.

Response
We have implemented the "radical" reorganized of the results as suggested and we believe the paper is now much more readable. We provide some background to these changes below; thank you very much for these suggestions.

Then, I would follow this with the more detailed "linkage" analyses of families 1-3 in which the various sex chromosomes are identified. Then, this could be followed by the RNA-seq expression analyses, which would now be much easier to understand because we know the sex chromosome genotypes of the parents of this cross. Finally, investigation of genome-wide recombination rates could come at the end.

Response
We agree and have reorganized the results as suggested.

I leave it to the authors to determine the optimal organisation of the manuscript, but at least some changes to the organisation and clarity need to be made, as the current order made things unclear for me and for the other two reviewers.

Response
We agree and have made many or all of the suggested changes; thank you for this very helpful feedback!

2. The presentation of the mapping results for Families 1 and 2 is still extremely confusing and hard to follow. Here is my interpretation of the data with a suggestion for a concise summary:

Five sex-linked RRGS markers were found in the region between 8.1Mb and 13.58Mb on chromosome 7 in Family 1, while in Family 2, three sex-linked RRGS markers were found in the region between 2.7Mb and 6.54Mb on chromosome 7. However, there were no informative RRGS markers between 6.54Mb and 11Mb in Family 2, so it was not possible to assess whether RGGS markers in this region were also sex-linked. Genotyping of additional markers in Family 2 by Sanger sequencing found three sex-linked markers located between 8.1Mb and 10.26Mb, suggesting that this region is sex-linked in both Families 1 and 2. [I know it is not perfect sex-linkage, which might require a little more explanation, but not much – just that recombination occurs outside the sex-determination region!].

By contrast, informative RRGS markers between 0 and 8.1Mb were present in Family 1, but were not sex-linked in this family. [then here you can use the discussion of reasons why found in L180-190, although this could be more clearly written]. Here you could also integrate the Sanger data for this family into the discussion…

Response
We have extensively reorganized this section and incorporated modfications of some of this suggested text; thank you!

Having a separate discussion of the Sanger data is just confusing and long-winded. As suggested above, I think it would be better to integrate the Sanger and RRGS data for each family/population, with perhaps the brief paragraph on the analyses of the Sanger data in the Nigeria strain (L212-219) at the end of this section. The table with the Sanger data could probably be Supplemental.

Response
We have shortened the discussion of the Sanger data in the Results section. We have opted to keep the Table in the main text. We refer to it several times and we feel it is a nice complement to the RRGS data.

Detailed comments: 1. L78: this sentence about the identity of master sex determining genes is not really connected to the rest of the paragraph. It could easily be deleted. If it remains, it needs to be clarified how it is related to the other information in this paragraph.

Response
We have deleted this sentence.

2. L82-85: this is an example of a long sentence with several spelling and grammatical errors, which could be shortened, perhaps to "Specifically, these transition periods may offer insights into whether and how characteristics of ancestral sex chromosomes (e.g. nucleotide divergence, sex-biased expression, degeneration [not degeneracy]) affect the evolution of the sex chromosome systems that follow."

Response
We agree and have made this change as suggested.

3. L141: "which can be combined in six ways for reproduction" is not correct, you are showing the possible offspring genotypes. Just say "and six possible offspring genotype combinations". [This new Figure 1 is super helpful!!!].

Response
We have changed this sentence to read "There are two possible sex chromosome genotypes in females (WZ, WW) and three in males (ZZ, ZY, WY), and six possible parental genotype combinations"

4. L155: "We intentionally sampled" makes it seem like a subset of the offspring were sampled for genotyping, but you are claiming that there are equal sex ratios in this

cross. Whether you genotyped all offspring in the cross, or a subset should be clarified. If a subset, you should give the total numbers so a reader can see whether there was an equal sex ratio in the cross.

Response
We have clarified that we sampled a subset of offspring from each family with approximately equal number of males and females. We did not quantify the exact sex ratio and do not claim that it was equal. Instead we point out that we (i) observed sons, and (ii) observed 100% male-linked SNPs. These two observations allow us to narrow down the parental genotypes to the unshaded options in Fig. 1.

5. Figure 2: Please add the Family names to the labels; i.e. Family 1 Ghana west, and Family 2 Ghana east, as you are referring to families in the text, making it challenging for the reader to connect the text and figure.

Response
We have added this label to this figure (which is now Fig. 4).

6. Figure 2 legend: As suggested by Reviewer 1, "Manhattan plot of sex linkage" is not really a clear description. Perhaps "Manhattan plot of association between genotype and sex phenotype on chromosome 7". Please delete "the" before Family 1. Also, I could not really see the difference in the dark and light dots between the top and bottom graphs, and this explanation in the legend "Dark and light dots indicate variants with a significant or not significant association with sex" seems to refer to the darker dots which have a strong association with sex, and the lighter dots on the rest of the chromosome. So, then the phrase "respectively, after FDR correction in (top) and before FDR correction in (bottom) is very unclear. And not all three shades of blue are shown in the top legend of the figure. This needs to be clarified.

Response
Thank you for these suggestions. We have made the change as suggested. We agree the description of the colors was difficult to understand and we have clarified this in the legend.

7. L221-222: Here it would be good to clarify how many individuals of each sex and population were used for the Fst analyses.

Response
We have added this information.

8. L232-234: The RRGS data from Family 2 only showed evidence for sex-linked markers between 0 and 6 Mb. Perhaps just say, "in the sex-linked region (between 0 and 11Mb) of chromosome 7 that was identified in the mapping families.

Response
In response to this suggestion and other suggestions below, we have

changed this to read “There were no informative RRGS markers between 6.54 Mb and 11 Mb on chromosome 7 of v10 in Family 2, so it was not possible to assess whether RGGS markers in this region were also sex-linked. However, genotyping of additional markers in Family 2 by Sanger sequencing found three completely or almost completely sex-linked markers located between 8.1 Mb and 10.26 Mb, suggesting that this region is sex-linked in both Families 1 and 2 (Table 1). By contrast, informative RRGS markers between 0 and 8.1 Mb on chromosome 7 of v10 were present in Family 1, but were not sex-linked in this family even though this region was sex-linked in Family 2.”

9. L234: by “genome-wide Fst peak” do you mean that the highest Fst value in the genome was found at this location?

Response
Yes. We have changed this to read “The highest Fst value in the genome (0.13) was present at position 9,940,000 in the sex-linked region of chromosome 7 in v10”

10. L240: “female-linked linkage group” could be “female-linked genomic region”.

Response
We have made this change as suggested and in parentheses prepended the names of linkage groups from Mitros et al. with “linkage group”

11. L241-245: this additional information about the overlap between these Sanger SNPs and the female-linked regions in the previous study does not seem necessary. You have already established that the regions of differentiation here are the same as in previous studies. If you do keep this text, please also change the “female-linked – linkage group” in line 242 to “female-linked genomic region” because it reads like it is a separate linkage group when you just mean that it is a separate region on chromosome 7.

Response
We have made this change as suggested and prepended the names of linkage groups as detailed above.

12. L245: you conclude this paragraph by saying the sex-linked region is between 0 and 10.4Mb [I agree with Reviewer 1 that using <10.4Mb is not very clear], but you started the paragraph by saying it was between 0 and 11Mb. Please be consistent here and throughout the manuscript. Or define that the region of high Fst was between 0 and 10.4Mb. Also, I think it would be better to start this paragraph by first stating the genome-wide Fst, then the average across the sex-linked region, and then finally the peak marker. This provides better context for the reader to see that the peak Fst of 0.13 is truly a peak.

Response

We have reorganized this section as suggested and now consistently refer to the upper limit of sex-linkage as being 10.4 Mb.

13. L255: perhaps provide the coordinates of the markers used for genotyping by Sanger sequencing.

Response
We have added this information and also added a reference to Table 1

14. L256-260: by moving the analyses of the nucleotide diversity in Family 3 earlier, you do not have to include this lengthy discussion as you would have already given the probable genotypes of this cross!

Response
This section has been reorganized but we have retained some of the description of possible offspring genotypes because we feel that it assists in the interpretation of the RNAseq results.

15. L264: clarify that these scaffolds were not part of chromosome assemblies

Response
Done.

16. L266-267: this first sentence could be deleted, as well as "for example" in L268

Response
Both suggestions were implemented.

17. L271: here you say the region of sex-linkage is less than 10.4Mb, and in L310, you say it is less than 11Mb. Another example of inconsistencies in the manuscript.

Response
We have corrected this to say <10.4 Mb throughout the manuscript.

18. L311-344: as suggested, this section could be moved earlier, and also shortened.

Response
We have shortened and reorganized this section.

19. L345-374: these points seem more relevant to the analyses of the sex chromosome complement of the crosses, rather than the analyses of gene expression. I think they could also be moved earlier, but also be shortened and/or be supplemental.

Response
We have moved this section to the Supplement.

20. L375-398: I really like these new analyses! It would seem to follow most naturally after the analyses of the male vs female RNA-seq analyses (especially if you follow the suggestion to move the analyses of nucleotide divergence earlier in the

manuscript). In these paragraphs, please also change "degeneracy" to "degeneration of".

Response
Thank you! We have also made this correction.

21. Figure 6: As in Figure 2, please label the graphs as Family 1 Ghana west, and Family 2 Ghana east.

Response
Done.

22. L407-408 and L412: "the size in base pairs of the genomic region to which the linkage map corresponds" is so awkward and wordy! Perhaps change to the "physical size in base pairs of the chromosome assembly". Also, here you say "females maps" and "males maps" instead of female and male maps.

Response
Thank you for these suggestions; they have been implemented.

23. L428: "subdivision is within" to "subdivisions are found within"

Response
Changed as suggested.

24. L443: "two localities Ghana" should be "two localities in Ghana"

Response
Corrected.

25. L446-447: how are your findings consistent with the origin of the Y in Nigeria? You found evidence for a Y in Nigeria, but it's not clear how that is evidence that it originated there.

Response
We have changed this sentence to read "However, the authors concluded that if there was a Y-chromosome, it would have originated from the Nigeria strain that was used in their cross [29]."

26. L448: It might be helpful to say where the Ivory Coast populations are located, relative to those shown in Figure 7.

Response
We agree; done.

27. L463-465: this sentence is hard to parse. Also, just because you found these chromosomes in your crosses, doesn't mean they are common. You might have just gotten lucky. Thus, I don't think you can say anything about whether they are on the brink of extinction.

Response

We agree and have changed this to read “Results presented here provide direct or indirect evidence that all three sex chromosomes were present in Family 2+3 and 1, respectively, but does not quantify the frequencies of each of these chromosomes in natural populations. Further efforts to genotype sex chromosomes of *X. tropicalis* sampled in nature could evaluate these possibilities.”

28. L499-501: less than “one hundred and fiftieth”: can you just give the percentage; i.e. less than 1% of the genome? And, it doesn’t seem necessary to give two different ways to characterize the abundance of sex-biased expression in this region of the genome. This is quite clear already.

Response
We have changed this to a percentage and deleted the second sentence

29. L503-506: there is an interesting paper by Jun Kitano’s group (Kitano et al. 2020 Journal of Evolutionary Biology doi: 10.1111/jeb.13662), suggesting that sex-biased expression at earlier stages of development is more likely to be due to sex-linkage, while sex-biased expression at later stages of development is more likely to be due to hormonal mechanisms. These results are also consistent with that hypothesis.

Response
Thank you for pointing out this reference. This is a very neat study! However, we do not think it provides an alternative explanation for this particular observation that we observed significant male-biased expression of sex-linked transcripts in juveniles but another study did not find a significant pattern in adults (although the trend was similar). under Kitano’s hypothesis, we’d expect a stronger signal in adults, where hormones and their receptors are fully expressed, than in juvenile tissues. However, we agree that this study is highly relevant and we have added this sentence and citation: “Sex-biased expression of sex-linked transcripts in multiple developmental stages has also been observed in fish, and may be a more effective mechanism for resolving genomic conflict in broadly expressed transcripts than differential expression orchestrated by steroid hormones [citation]. ”

30. L517-518: I don’t quite understand the point here.

Response
This sentence has been changed to read “However, in *X. tropicalis* this scenario does not appear to apply since the degenerate W-chromosome is staged to survive a transition to male heterogamy if the Y-chromosome fixes in the future because it would become an X-chromosome”

31. L519-521: this sentence doesn’t seem necessary, but the faster-Z should probably be briefly explained in the next sentence.

Response
We deleted these two sentences.

32. L541: “each one generation in length” could be deleted.

Response
Done.

33. L570: “evidence W-chromosome degeneracy” could be “evidence for W-chromosome degeneration”.

Response
Done.

34. L589-591: I think this information comes later, but it would be good to provide the numbers of wild-caught males and female samples in each population used for RRGS.

Response
Done.

35. L613-614: “or for the offspring” seems incomplete.

Response
This was a typo that has been deleted.

36. L619: “east” should be “Family 2” for clarity.

Response
Changed as suggested.

37. L631-633: in the absence of providing any details about these markers, there should at least be a reference the Table with this marker information.

Response
A reference to Table 1 has been added.

38. L650: “likelihood of their being a SNP” should be “likelihood of there being a SNP”

Response
Corrected.

39. L665-666: it would be good to provide the genomic location of these markers used to genotype the tadpoles.

Response
This is now done earlier in the Results and a reference to Table 1 has been added here as well.

Reviewer’s Responses to Questions

Comments to the Authors: Please note here if the review is uploaded as an attachment.

Reviewer 1: The manuscript is improved by the extra analysis, but it remains poorly written and largely descriptive of a further study of a case of within-species variation in the heterogametic sex. Much of the text is long-winded and difficult to understand, obscuring the main points. It is valuable to get genetic data, and this appears to establish that there is no physically extensive fully sex-linked region in the species studied, consistent with its apparently homomorphic sex chromosomes, and with the fact (already known) that it has a sex chromosome polymorphism with Z, W and a more recently derived Y chromosomes, similar to the polymorphisms in some other frogs.

The study may indeed have identified, for the first time in natural X. tropicalis population samples, the Y-chromosome previously found in captive individuals. The paragraph starting in line 442 is very long, and not easy to follow. The information needs to be digested and presented more clearly after the authors have decided what they need to tell readers.

> **Response**
> This paragraph has been shortened slightly by the shortening of the 3rd and penultimate sentences.

I hoped for an advance in understanding, or at least a clear discussion, of whether the results support one model for turnover events or another, but it is not clear to me what these results tell us about turnovers.

> **Response**
> We feel that our study tells us several things about sex chromosome turnovers. We demonstrate that the Y-chromosome evolved from an ancestral Z-chromosome and not from an ancestral W-chromosome. We also show that degeneration of a small portion of the W chromosome caused a high concentration of transcripts with sex-biased expression and that this bias lingers on through intermediate stages of sex chromosome transition. These two results are important to the field and with few similar empirical examples in the literature (none for the second point that we are aware of). We also wish to point out that this study is not only about sex chromosome turnovers - it also provides new insights into the nature of genome-wide recombination rates and population structure in this widely studied species. Finally, we also show that the male determining locus on the Y chromosome is in a very similar location as the female determining locus on the W chromosome. This is also a completely novel finding that suggests that variants at the same gene may drive sex-specificity of the sex-linked portions of the W and Y chromosomes in females and males respectively.

The most interesting other result is that there are unexpectedly many transcripts with significantly male-biased or (not "and" as written in line 278) male-specific expression on chromosome 7.

> **Response**
> We have corrected this as suggested.

I do not have the necessary expertise to evaluate this, but obviously it is important to use reference genome from an appropriate sex in order to avoid a bias due to potential absence of some sequences in some fully sex-linked regions. The paper by Wei and D. Bachtrog, 2019 is now cited, but this problem, and the reference assemblies used, should surely be mentioned in the main text, not just a Supplementary file.

> **Response**
> We have added this text to the Methods Section "The analysis of RRGS data involved mapping reads to a reference genome that was generated from a female individual of unknown sex chromosome genotype. Possible concerns with and justification of this approach are discussed in further detail in the Supplement."

The Discussion (from line 495) deals with this finding as a "Sign of Age in Cytologically Indistinguishable Sex Chromosomes", but I think the authors probably mean "sign of adaptation" or maybe "sign of genetic degeneration of the W". Again the text could be clearer after shortening to make the essential point(s) clear. In the present version, the evidence for the claimed genetic degeneration of the W chromosome is not compelling, and needs to be much clearer.

> **Response**
> We have changed the section title to "Signs of Genetic Degeneration in Cytologically Indistinguishable Sex Chromosomes" as suggested. We feel that the evidence for sex chromosome degeneration are compelling, as evidenced by (i) a high number of genes with male-biased expression in the sex-linked region and (ii) a much higher level of divergence in sex linked transcripts in individuals with putative WZ, WY, and ZY genotypes compared to individuals with WW genotypes. We agree that further study of sex chromosome degeneration would be valuable, but feel as well that we have made significant advances in understanding of this system, including providing convincing evidence for sex chromosome degeneration in the small sex-linked region of this species' genome.

The following text about these expression results is again very hard to understand, but may mean the following: We therefore examined the genotypes in sex-linked expressed transcripts of each individual offspring of Family 3 using the RNAseq data, in order to detect transcripts expressed from only one of the individual's sex chromosomes (based on observing no heterozygous variants), and those co-expressed by both gametologous alleles in heterozygous genotypes [17]. I leave the rest of this section for

the authors to revise and clarify.

Response
We have implemented this suggestion and removed the long winded sentence that followed.

It is very confusing to write about "levels of polymorphism" when genotypes are meant. Possibly a table showing the possibilities (including a degenerated W-chromosome), and the expectations in the different situations and the different parental genotypes, would be helpful. Ideally the results would be digested and shown as a comparison with those expectations, so that the text can guide the reader to understand the evidence for the results about the sex-specificity of rates and locations of recombination in X. tropicalis are potentially helpful for understanding how and/or why a turnover happened, though I still found the conclusions rather weakly supported. The results are consistent with the conclusion, but not compelling. The discussion of what we can learn from this is improved, but still appears not to be related to the claimed genetic degeneration of the W chromosome.

Response
Thank you for these constructive comments. We changed the term"levels of polymorphism" to "nucleotide diversity". As discussed in a previous response, we feel that the nucleotide diversity analysis coupled with high concentration of transcripts encoded by sex-linked genes does argue strongly for genetic degeneration, although we agree that further exploration of this issue would be exciting as well.

The section about population structure needs an introduction to give an idea of why these data are needed. This could maybe be merged with the start of the Discussion.

Response
We have moved the section on population structure to the beginning of the Results. In the revision we have not altered the beginning of the Discussion which has a brief introduction to the geographical setting of this species.

MINOR COMMENTS

The writing still needs considerable revision, as it is often very long-winded, making it difficult to understand the meaning.

Throughout, the manner of specifying regions on a chromosome like this example "<10.4Mb" should be changed to be clear to readers.

Response
Done.

There should also be no hyphens in the phrase Y (or other) chromosome.

Response
We have removed all instances of hyphenated sex chromosomes.

The authors should read the text carefully for errors (I have not listed all of them here), and make sure that the word “that” is not missing in places where it is needed in written English.

Response
Done.

SHORTENINGS AND EDITINGS Line 67: Shorten to: A sex chromosome turnover is called "homologous" when a new variant that assumes the sex determination role arises on an ancestral sex chromosome [1], and "non-homologous" if it establishes a on a different chromosome pair from the ancestral sex chromosomes.

Response
Thank you for these suggestions; we have made the suggested changes to these sentences although we have retained our original citations.

Line 74 Turnovers 9in the plural)

Response
Changed.

Line 75:. Non-homologous XY to XY turnovers may be favoured by natural selection if the ancestral Y-chromosome has a high load of deleterious mutations due to genetic degeneration [21,22]. However, Y-linked deleterious mutations may disfavour XY to WZ transitions because they result in the appearance of homozygotes for the ancestral Y-chromosome[16].

Response
We have made this change as suggested; thank you!

Surely a new paragraph is needed before “So far, only a handful of master sex determining genes are known”.

Response
This sentence was deleted in response to a previous suggestion and we have also started a new paragraph here.

Line 82 Specifically, these transitions [should be singular] periods may offer insights . . . .

Response
Corrected.

Line 95: Although it is technically no longer a Z-chromosome after the Y-chromosome appeared, we use this term as a placeholder to refer to the extant non-male-specific sex-chromosome that descended from 97 the ancestral Z-chromosome,

following [28]" can be shortened to "We use the term 'Z-chromosome', following [28]', to refer to the extant non-male-specific sex-chromosome that presumably evolved from the ancestral Z-chromosome still present in related species". Is this the correct meaning? How is it known that this Z is the ancestral one, and that the Y is derived? If the evidence is in this paper, why not make that clear here?

> **Response**
> This is not a research finding – we simply wish to clarify that we are calling the extant descendant of the ancestral Z chromosome a Z chromosome, even though its mode of inheritance is not exactly like a Z chromosome because there are three sex chromosome segregating concurrently. We have slightly restructured this sentence in hopes of making it clearer.

Line 102: The genomic location of the female-associated region of the W-chromosome in a laboratory strain was narrowed down [BY WHAT APPROACH?] to an interval between positions 0–3.9 megabases (Mb) on chromosome 7 in genome assembly 9.1 (v9) [30]. However, this region did not show complete linkage to the female phenotype in our study (below), and it was proposed that this could be due to ancestral admixture with an individual carrying a Y-chromosome [30]. The male determining factor of the X. tropicalis Y-chromosome of is thought to be in a similar location to that of the female-determining factor [28], but has nor been precisely located. Within the genus Xenopus, the most recent common ancestor of subgenus Silurana, which includes X. tropicalis, probably had heterogametic females [26], implying that the X. tropicalis Y chromosome is derived from an ancestral W or Z chromosome. Mitochondrial genomes of species in subgenus Silurana diverged about 25 million years ago [33], implying that the Y-chromosome of X. tropicalis is younger than that. This variation raises the possibility that X. tropicalis is currently undergoing a homologous sex chromosome turnover. HERE IT IS NOT CLEAR WHAT 'variation' IS BEING MENTIONED.

> **Response**
> We have clarified this section to stipulate that it was the study of Mitros et al. (not ours) that failed to find completely sex-linked sites and that they used genetic mapping to identify this region. We also changed the term "variation" to "information".

Line 122: We also evaluate whether and how recombination differs between the sexes of X. tropicalis – including in the sex-linked region and across the genome. The goals of this study are to . . . .

> **Response**
> Changed as suggested; thank you

Line 131 : a comma is needed before "and their offspring"

> **Response**
> Added.

Line 135: We also studied sex linkage in different portions of the sex-linked region, using Sanger sequencing of selected amplicons.

Response
Changed as suggested.

Line 140: There are two possible sex chromosome genotypes in females (WZ, WW) and three in males (ZZ, ZY, WY), and six possible parent genotype combinations (Fig. 1).

Response
Changed as suggested.

Line 170: None of the sex-linked SNPs from Families 1 and 2 were present in the sequences of the other family, presumably reflecting variable presence of SbfI restriction sites. In each of the Ghana families, some SNPs in the region that was sex linked in one family displayed genotypes suggesting independent segregation in the other (Fig. 2). One possible explanation is that our sex-linked markers are partially sex-linked. In Ghana East Family 2, we lacked markers between 6-11Mb on chromosome 7, and therefore our the RRGS data cannot delimit the fully sex-linked region In this family. THE QUESTION IS WHETHER THESE MARKERS ARE PARTIALLY SEX-LINKED OR SEGREGATE INDEPENDENTLY OF THE SEX-DETERMINING LOCUS.

Response
Thank you for these helpful suggestions. We have implemented most of them. As discussed in the main text, the discrepancies between the families are a consequence of (i) non-overlapping markers in each family and also (ii) different sex chromosome genotypes of the father of each family (putatively Zy in Family 1 and putatively WY in family 2). We have attempted to clarify these conclusions throughout this revised manuscript, including in the section addressed in the next comment.

Line 179: The writing needs correcting here (the current version reads rather raw and undigested) — "On chromosome 7 <6Mb, we had variable markers that overlapped in Family 1 and 2, and these markers were male-linked in Family 2 but not Family 1 (Fig. 2). Analyses discussed below allowed us to infer. . . .This scenario would explain why there were sex-linked sites on the end of chromosome 7 in Family 2 but not Family 1; it is also consistent with evidence presented below for a lack of recombination in the sex-linked region during spermatogenesis of male BJE4362, the father of families 2 and 3, and with an origin of the Y chromosome from an ancestral Z chromosome

Response
We have shortened this section and incorporated these suggestions; thank you!

Figure 2 legend: I don't think one can just sat "Manhattan plot of sex linkage for

chromosome. . . ."

Response
We agree and have changed this legend as also suggested by the Associate Editor.

Line 193: Sanger sequencing identified one SNP that co-segregates in our small Family 2 with the sex-determining locus, and two almost completely co-segregating SNPs. With a very small sample size we identified three completely co-segregating SNPs in the Ghana east wild population, but none in either Family 1 or the Ghana west wild population. The lack of sex-specific SNPs (even with our small sample) in Family 1 is consistent with the hypothesis proposed above that the sex chromosome genotype of the father may have the ZY genotype (though other explanations are possible).

Response
We have changed this text as suggested; thank you.

Line 216: However, without invoking Y linkage, the chance of observing a heterozygous genotype in 17 of 17 males and none of 18 females is very low ($_{71}$06).

Response
We have made this change as suggested.

This sentence is hard to understand, and so is its relevance (and the tense is wrong): "The sex-linked SNPs in the Nigeria strain was different from a nearby sex-linked SNP in three wild Ghana east males."

Response
We changed this sentence to read "The sex-linked SNPs in the Nigeria strain are in different genomic positions from the nearby sex-linked SNP in three wild Ghana east males."

Line 220: The X. tropicalis Sex Chromosomes Have ONLY a Small Differentiated Region

Response
We combined this section with the section on population structure and have changed the subheading to reflect this: "Population structure in *X. tropicalis* and a Small Region of Sex Chromosome Differentiation".

Line 223: This passage is particularly badly written and can be much shorter and clearer. As demonstrated above, some of these individuals have a Y chromosome, and , because a W chromosome is required for female development, they must all have a W; some individuals of either sex may have a Z chromosome along with their Y or W. Differences in allele frequencies are expected to affect FST between females and males, and nucleotide divergence between the Y, Z and W chromosomes leads to different frequencies in the two sexes, and can reveal fully sex-linked regions, with higher differentiation than elsewhere in the same chromosome.

Response
This section has been shortened and modified in part as suggested: " We then quantified Fst between females (n = 12) and males (n = 26) over a moving average of 50 SNPs in wild individuals from Ghana, and georeferenced lab individuals from Sierra Leone and Nigeria. Population structure coupled with different geographic sampling of males and females should have a genome-wide effect on Fst between females and males. There are two possible sex chromosome genotypes in females (WZ, WW) and three in males (ZZ, ZY, WY), and six possible parental genotype combinations (Fig. 2). Therefore, in sex-linked regions, differences in allele frequencies and nucleotide divergence between the W, Y, and Z chromosomes are expected to cause Fst to be higher than elsewhere in the genome, including pseudoautosomal regions of the sex chromosome."

We indeed observed higher FST values in the region where sex-linkage was detected in our analyses above using RRGS data from Family 2 (the chromosome 7 region before 11Mb), with an FST value of 0.13 at position 9,940,000 in the v10 assembly of this chromosome, and values >0.09 between positions 9,775,600 and 9,999,600 (1,615,479 to 1,454,645 – 1,664,477 in assembly v9). The genome-wide 95% CI is 0.002 – 0.038. [THIS SHOULD BE SPECIFIED HERE, NOT LATER]. Are the high FST values due to low diversity within the population of X (or other relevant) chromosomes?

Response
This section has been extensively revised in response to this suggestion and one of the Associate Editor.

Line 239 : should be changed to "found previously [30] (Suppl.Fig. S3)."

Response
Changed as suggested.

Line 266: This sentence is not clear (and can probably be omitted). For neutral variants in an autosomal locus, genetic drift of the transcriptome is not expected to produce a skew in sex-biased expression.

Response
We agree and have deleted this sentence.

Line 304" another problem with tenses — "it does suggest that there is no reason to expect that transcripts in this genomic region was somehow predisposed to have male-biased expression"

Response
We agree and have changed "was" to "are".

Line 307: There is no need to keep repeating the information that high densities of male- or female-biased expression are rare on the autosomes. This is repeated 3 times

in quick succession.

Response
We have deleted two sentences that were repetitive in this regard.

Line 311: section on How might sex-linkage lead to a skew towards male-biased expression of transcripts encoded by sex linked genes? Again, the writing is vague and unclear. I think the first sentence (One possible explanation is that expression of some alleles in the sex-linked region of the chromosome decreased or was [wrong tense again] lost due to recombination suppression) is referring to genetic degeneration, but it is hard to be sure if this is the meaning. Maybe it is supposed to mean "One possibility is that expression of some alleles in the sex-linked region of the chromosome became decreased below the level of alleles on an ancestral non-degenerated Z-chromosome, and thus alleles that are now Y-linked (and were derived from a Z) retain the Z's high ancestral expression level".

Response
This sentence has been removed and this section was extensively revised.

Line 432: "interceded" is the wrong word here.

Response
We have changed "interceded" to "interrupted".

Thank you very much for these helpful comments; I sincerely appreciate the time that you spent providing this constructive feedback on this revision and also the initial submission.

Reviewer 2: I think that the current version of the paper is much stronger - the analyses and interpretations are sound, and the results still very exciting. The authors did a great job at addressing my previous comments (although I would specify "but only a single female and 1 to 3 males were sequenced" rather than saying "with a very small sample size").

Response
Thank you for this suggestion; we have added this clarification to the text.

My only remaining comment is that I still sometimes found the manuscript a bit hard to follow (but figure 1 is very helpful!).

Response
This was also a key concern of the other reviewer and the Associate Editor. We have performed an extensive revision of the structure of the Results to address this concern.

Some of that seems to come from the fact that the structure of the paper reflects its history. I think it would make more sense to have the inference of the genotypes of

the three families in the first section (including the RNA), and then going into the gene expression knowing those genotypes, instead of going back to them later.

Response
We have extensively revised the Results section to present information in a conceptual rather than historical order. For example, the analysis of nucleotide variation in the RNAseq data is now presented with the RRGS linkage analysis (which is also based on nucleotide data) and the expression data is separately presented after this.

In general the text seems (to me) a bit longer than necessary. For instance, the Sanger sequencing part, from which not much was concluded, could maybe be shorter or even moved to the supplementary material.

Response
We have shortened the paper in several places by deleting text or moving it to the Supplement. But we opted to retain the Sanger sequencing results in the main text because we felt it added important context. Thank you very much for your comments on this revision and the previous submission.
